# Isotopic evidence for volatile replenishment of the Moon during the Late Accretion

**DOI:** 10.1093/nsr/nwz033

**Published:** 2019-03-11

**Authors:** Yanhao Lin, Wim van Westrenen

**Affiliations:** Department of Earth Sciences, Vrije Universiteit Amsterdam, Amsterdam 1081 HV, The Netherlands

**Keywords:** hydrogen and chlorine isotopes, lunar apatite, volatile replenishment, Late Accretion

## Abstract

The traditional view of a dry, volatile-poor Moon has been challenged by the identification of water and other volatiles in lunar samples, but the volatile budget delivery time(s), source(s) and temporal evolution remain poorly constrained. Here we show that hydrogen and chlorine isotopic ratios in lunar apatite changed significantly during the Late Accretion (LA, 4.1–3.8 billion years ago). During this period, deuterium/hydrogen ratios in the Moon changed from initial carbonaceous-chondrite-like values to values consistent with an influx of ordinary-chondrite-like material and pre-LA elevated δ^37^Cl values drop towards lower chondrite-like values. Inferred pre-LA lunar interior water contents are significantly lower than pristine values suggesting degassing, followed by an increase during the LA. These trends are consistent with dynamic models of solar-system evolution, suggesting that the Moon's (and Earth's) initial volatiles were replenished ∼0.5 Ga after their formation, with their final budgets reflecting a mixture of sources and delivery times.

## INTRODUCTION

**Figure 1.**
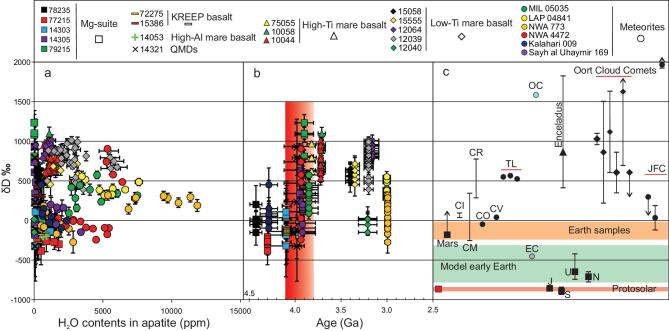
Compilation of hydrogen isotopic composition, water content and crystallization ages in lunar apatite. Deuterium/hydrogen (D/H) ratios, reported using δD values, with δD = {[(D/H)_sample_/(D/H)_VSMOW_] – 1} × 1000 (VSMOW: Vienna standard mean ocean water), are shown versus measured OH abundances in ppm (a) and versus sample crystallization age (b). The red bar represents the Late Accretion. Panel (c) shows the hydrogen isotopic composition of major solar-system reservoirs including Earth samples; model early Earth at the time of Moon formation; Mars, Jupiter (J), Saturn (S), Uranus (U) and Neptune (N); Saturn's Moon Enceladus; chondrite groups carbonaceous chondrites groups CI, CM, CR, CO and CV; Tagish Lake (TL) samples 11i, 11h and 5b; Oort Cloud Comets 1P/Halley, Hyakutake, Hale-Bopp, C/2002 T7 (LINEAR), 8P Tuttle and 153P/Ikeya-Zhang, respectively; and Jupiter-family comet (JFC). Arrows indicate δD values beyond the maximum or minimum of the *y*-axis scale. Error bars are 2σ. See Supplementary Tables 1 and 3 in the Supplementary data, available as Supplementary Data at *NSR* online, for all data and references.

**Figure 2.**
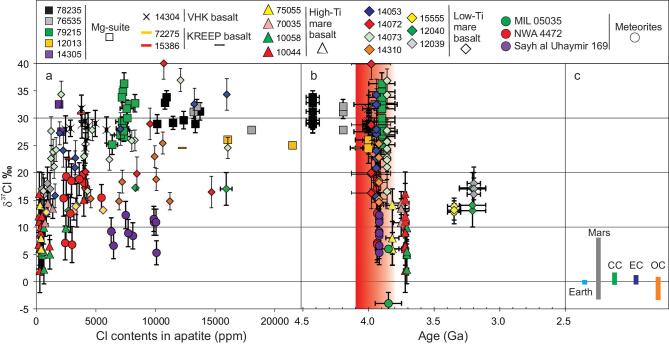
Compilation of chlorine isotopic composition, chlorine content and crystallization ages in lunar apatite. ^37^Cl/^35^Cl ratios, reported using δ^37^Cl values, with δ^37^Cl = {[(^37^Cl/^35^Cl)_sample_/(^37^Cl/^35^Cl)_SMOC_] – 1} × 1000 (SMOC: standard mean ocean chloride), are shown versus measured Cl abundances in ppm (a) and versus sample crystallization age (b). The red bar represents the Late Accretion. Panel (c) shows the chlorine isotopic composition of major solar-system reservoirs including Earth; average of carbonaceous chondrite (CC) groups CI, CM, CR, CO and CV; enstatite chondrites (EC) and ordinary chondrites (OC). Error bars are 2σ. See Supplementary Tables 2 and 3 in the Supplementary data, available as Supplementary Data at *NSR* online, for all data and references.

The abundance and isotopic composition of hydrogen and chlorine in apatite provides one of a limited set of windows into the origin and history of the volatile budget of the Moon. Many studies have reported high-precision apatite volatile abundance and isotopic measurements from both lunar meteorites [1,2] and Apollo samples [1–13]. Typically, these studies focus on identifying and explaining variations among and between distinct petrological groups (e.g. low-titanium and high-titanium mare basalts, KREEP (enriched in potassium (K), rare-earth elements and phosphorus (P)) basalts, and highland samples) in terms of igneous processes including fractional crystallization, degassing and mixing. To date, such measurements have not been discussed in terms of variations as a function of sample age. Figures 1a and 2a present a compilation of >260 H and >120 Cl isotopic and abundance measurements published over the past 8 years. All data sources and measurements are presented in the Supplementary data, available as Supplementary Data at *NSR* online; note that, for most samples, either hydrogen or chlorine isotopes were measured. Deuterium/hydrogen (D/H) ratios are reported using δD values, with δD = {[(D/H)_sample_/(D/H)_VSMOW_] – 1} × 1000 (VSMOW: Vienna standard mean ocean water). ^37^Cl/^35^Cl ratios, reported using δ^37^Cl values, with δ^37^Cl = {[(^37^Cl/^35^Cl)_sample_/(^37^Cl/^35^Cl)_SMOC_] – 1} × 1000 (SMOC: standard mean ocean chloride). Sample ages are predominantly whole-rock ages, and we assume that the ages plotted in Figs. 1 and 2 reflect the ages of the apatites themselves.

## RESULTS

### Hydrogen isotopic shifts during the LA

Plotting all individual measured isotopic compositions of samples as a function of their independently determined formation age (Figs. 1b and 2b) reveals previously undetected and striking changes in apatite isotopic composition through time, centered on ∼4.1–3.8 Ga. In the lunar literature, this period is variously referred to as the Late Heavy Bombardment (LHB) period, the final episode or Terminal Cataclysm at the end of the LHB, or the final stage of the Late Accretion (LA) [14–21]. In this paper, we refer to this period as the LA. Pre-LA δD values in apatite are uniformly negative, with a δD value of −151 ± 74‰ (based on the Mg-suite samples and sample 72275 shown in Fig. 1b). As shown in Fig. 1c, this value is indistinguishable from the terrestrial [22] and carbonaceous chondrites [23] values and slightly higher than the hydrogen isotopic compositions of enstatite chondrites [24]. Post-LA apatites from high-Ti mare basalts record an average δD of +763 ± 47‰, followed by a minor decrease around ∼3.2 Ga. During the LA, variability in apatite δD is high and varies between pre-LA and post-LA averages. The process of hydrogen isotope fractionation during late-stage hydrogen degassing has been proposed to interpret high δD values in apatites [1,9]. However, as discussed by previous studies [7], the absence of a negative correlation between OH abundances in apatite and their δD (Fig. 1a) shows that degassing is not the source of the trend seen in Fig. 1a. Additionally, as discussed in the following section, late-stage degassing is also inconsistent with Cl isotopic data.

Alternatively, this increase can be explained by the addition of deuterium-rich material to the Moon during the LA. Addition of both ordinary

chondrites and comets could lead to an increase in δD (Fig. 1c) from pre-LA to post-LA values, the large variability during the LA reflecting variable mixing between the indigenous and added reservoirs. The positive correlation of δD in apatite vs. pyroxene Mg#_max_/Mg#_min_ for lunar basalts, previously ascribed to hydrogen incorporation from the regolith [25], could also be explained in this way. This correlation is consistent with δD values representing mixing between isotopically heavy and isotopically light components, rather than representing degassing trends.

Lunar siderophile element abundances [15,26], as well as solar-system dynamic models describing sudden changes in the orbits of the giant planets at the time of the LA [27,28], suggest that asteroids (as opposed to comets) dominated the LA impactor population, with Kring and Cohen [15] claiming ordinary chondrites (OC) and/or enstatite chondrites (EC) could provide the main source of LA impacts. Figure 1 shows that water addition by EC with a low-δD value of −460‰ [24] cannot explain the observed shift in δD. Figure 1 therefore suggests that OC, with high-δD (∼1620‰) [23] and relatively low water content (∼1.1 wt.%) [23], were the dominant impactors during the LA.

### Chlorine isotopic shifts during the LA

Pre-LA Cl isotopic measurements are also uniform, with δ^37^Cl = 30 ± 1‰. These values are significantly higher than the range of −4 to 0‰ found in Earth and chondritic meteorites (Fig. 2c). This could reflect significant Cl degassing in the period between the formation of the Moon and the formation of the oldest apatite sample for which Cl isotope data are available. The LA period is characterized by large variations in Cl isotopic composition, transitioning down to ∼8 ± 2‰ (from four samples formed close to the end of LA). Boyce *et al*. [2] observed a correlation between the δ^37^Cl in apatites from a set of post-LA lunar basalts and enrichment through the addition of a KREEP component, characterized by elevated La/Lu ratios and Th contents of the bulk rocks in which these apatites formed. Their model suggests that degassing during the (pre-LA) lunar magma-ocean (LMO) stage led to the formation of an isotopically heavy KREEP reservoir, which is tapped to various degrees by the post-LA samples. However, when pre-LA Mg-suite data are added, there is no clear correlation between δ^37^Cl and KREEP signature: pre-LA samples show the highest δ^37^Cl but have low Th and only moderately elevated La/Lu ratios (Supplementary Fig. 1 in Supplementary data, available as Supplementary Data at *NSR*

online). The Boyce *et al.* [2] model cannot explain the high pre-LA δ^37^Cl data seen in Fig. 2b and is also inconsistent with D/H ratios, which show a decrease with increasing KREEP influence as opposed to the expected increase. Instead, the data support the addition of chondritic material to the Moon during the LA, with variability during the LA attributed to variable mixing between the indigenous and added sources.

Our interpretation is consistent with studies of volatile abundances and isotopic ratios in lunar volcanic glass beads [29,30]. Such beads show up to 98% or more degassing of water [29] during eruption (i.e. during the transport of individual small blobs of melt through space). After correcting for degassing, these studies conclude that the sources of post-LA glass beads (amongst the most primitive samples on the Moon) have terrestrial (low) D/H ratios [29,30]. Our model is consistent with this, because we invoke the presence of two sources of volatiles in the Moon—one indigenous, the other added during the LA. The LA stage is reflected isotopically most clearly in samples formed during and shortly after the LA, but it is unlikely that the later source mixed fully with the original pre-LA source throughout the Moon. After the LA, both hydrogen and chlorine isotopic data seem to show a trend towards pre-LA values, reflecting melting from a source that is more dominated by the pre-LA values (more in line with the primitive LMO-processed mantle without the addition of external components). We reiterate that invoking degassing to explain all elevated D/H ratios shown in Fig. 1b severely complicates explanations for the coeval change to lighter Cl isotopic ratios shown in Fig. 2b.

Combined, Figs. 1 and 2 thus provide isotopic evidence for the addition of OC material to the Moon during the late heavy bombardment. The fact that this OC component is measurable in volcanic samples implies LA impactors were capable of breaching the primitive lunar crust and mixing (albeit certainly not perfectly) with at least part of the source regions of mare volcanism.

### N isotopic evidence

Additional evidence for the addition of volatiles to the Moon during the LA comes from lunar indigenous N isotope measurements. Isotope ratios of nitrogen (^15^N/^14^N) are expressed in the delta (δ) notation, where δ^15^N = {[(^15^N/^14^N)_sample_/(^15^N/^14^N)_std_] – 1} × 1000, in ‰, and the standard is terrestrial atmospheric N_2_ with ^15^N/^14^N = 0.003676. Recent studies show that several lunar samples that formed during and after the LA contain an indigenous heavy nitrogen isotopic reservoir with a range of δ^15^N from −7.6 ± 4.1 to 43.9 ± 2.8 at an average of 13 ± 7‰ [31–33]. This is isotopically heavier than nitrogen in Earth's depleted mantle (δ^15^N_mantle_ ≤ −30 to −5‰) [34]. Although this has previously been explained through degassing processes, it is also in agreement with the notion that ^15^N-rich materials provided volatiles to the Moon during the LA. EC can be excluded as a major source of LA impactors in this scenario, based on their δ^15^N of −29.2 ± 0.6‰ [35,36]. Saal *et al*. [29] previously pointed out that the nitrogen budgets of comets, quite distinct from the Moon, greatly limit the possible cometary contribution to the Moon. In contrast, OC (with a published δ^15^N of −18 to +95‰) [37] again provide a realistic LA material source.

## DISCUSSION

### Effect of shock-induced resetting of ages and/or isotopic compositions

Partial or full resetting of apatite isotopic data by events postdating the formation age of their host rocks cannot be fully excluded as, to date, none of the individual apatite grains for which H and Cl isotopic data have been obtained has been dated. However, several studies have provided phosphate U–Pb or Pb–Pb ages for separate grains in the same samples for which H and Cl isotopic data have been published (e.g. lunar meteorites LAP 02205 [38] and Kalahari 009 [39,40]). Apatite ages in these samples show no evidence of impact resetting after formation. In the absence of evidence to the contrary, we therefore assume that (i) whole-rock ages are a good indicator of phosphate ages and (ii) none of the H and Cl isotopic compositions of lunar apatite that we compiled was significantly affected by post-formation alteration processes.

### Constraining the size of the added reservoir

It is not possible to obtain quantitative information about the volatile content of lunar-rock source regions from volatile abundance measurements in apatite only, due to complexities in the apatite–melt exchange reactions involved [41,42]. In addition to apatite volatile abundance measurements, the chlorine content of the source rock in which the apatite formed is also required to derive source-rock volatile contents. Bulk lunar-rock chlorine measurements for apatite-bearing samples have not been made to date [42], but Hauri *et al*. [43] noted that Cl/Nd and Cl/Ba ratios in primitive lunar samples are constant, enabling estimates of source-rock chlorine contents using published bulk-rock Nd and Ba contents (see the ‘Methods’ section). Using this approach, we estimated bulk-rock Cl contents of pre-LA KREEP basalt 72275 (∼4.1 Ga) and three post-LA high-Ti mare basalts. Combining these data with the published *K*Ap-Melt dOH–Cl of 0.06 ± 0.02 [42] yields pre-LA source-rock water contents of ∼1.3 ppm and post-LA source-rock water contents of ∼33 ppm based on Cl/Nd. Cl/Ba-based calculations indicate ∼0.9 ppm water in the lunar interior pre-LA versus ∼15 ppm water post-LA, reasonably consistently with the Nd-based estimates (detailed calculations are provided in the ‘Methods’ section).

Experiments simulating the solidification of the LMO in the presence of water, combined with observations of lunar crustal thickness, suggest the Moon contained at least 270 ppm water during the crystallization of its magma ocean [43], consistently with the ∼320 ppm estimated by Hui *et al.* [44], namely before any of the apatites considered in our compilation formed. Our compilation suggests that, by the time the oldest apatites shown in Figs. 1 and 2 formed, the lunar interior only contained ∼1 ppm water, pointing to significant degassing of the Moon in the period between LMO crystallization and the formation of the apatites in the current database. The increase in water content from ∼1 ppm pre-LA to 15–33 ppm post-LA again suggests that significant amounts of volatiles were added to the Moon during the LA.

Using average pre-LA δD values of ∼ −150‰ and post-LA δD values of ∼ +760‰ based on our data compilation and an average OC composition containing 1.1 wt.% H_2_O and a δD value of ∼ +1620‰ [23], a simple two-component mixing model based on mass balance can be used to constrain the OC mass added during the LA (see the ‘Methods’ section). Ignoring loss through degassing on impact, the addition of 0.06–0.11 wt.% lunar mass is required to explain the hydrogen isotopic shift. Here, the lower bound assumes the post-LA water content is representative of the top 400 kilometers of the lunar interior [8] and the upper bound assumes the post-LA water content represents the whole silicate Moon. The result is essentially independent of the pre-LA lunar interior water content, because of the strong leverage provided by the OC composition. This 0.06–0.11 wt.% addition in terms of mass is 3–5 times larger than currently estimated from highly siderophile element (HSE) abundances in the lunar mantle [45,46], >10 times lower than expected based on scaling current estimates of the late mass addition to Earth derived from HSE abundances in the terrestrial mantle [47] and overlaps with estimates (0.1–0.4 [48] and <0.02–0.4 wt.% [49] of the lunar mass) based on previous models of lunar volatile abundances. The difference between HSE-based and volatile-based estimates could be due to incorrectly assuming sulfides play no significant role in controlling mantle HSE budgets [50]. Because OC contain <200 ppm Cl [51], the increase in lunar mantle source Cl due to the addition of 0.06–0.11 wt.% lunar mass OC material during the LA would be in the order of 0.2 ppm only.

### A low D/H ratio in the early Earth and the young Moon

Before the LA, the oldest samples for which data are available show low D/H ratios, close to those found in terrestrial samples (Fig. 1b and c). This appears to be contrary to the suggestion of early significant water and chlorine losses accompanying extensive degassing during the Moon's magma-ocean stage. If extensive degassing occurred during the magma-ocean stages, this implies that the Moon, when it formed, should have been characterized by a D/H ratio that is significantly lower than that found in lunar or terrestrial samples. This lends support to the recent model of Sharp [52], who proposes that the early Earth could have been characterized by a significantly lower D/H ratio than that of the present-day Earth, because of early ingressing into Earth of solar (extremely low) D/H ratio hydrogen (Fig. 1c). Although the exact D/H ratio of Earth at the time of the Moon formation remains unquantified, this provides a viable explanation for Earth-like D/H combined with heavy Cl isotopic composition for the old Mg-suite samples in Figs. 1 and 2: both can be explained by degassing in the early Moon during the magma-ocean stage. In this process, D/H is elevated back to terrestrial-like values from an initial low δD, at the same time elevating δ^37^Cl of the Moon.

## CONCLUSION

Our analysis indicates that the lunar interior volatile cycle is not characterized solely by progressive volatile loss from an initially volatile-rich body. Our analysis suggests that the initially substantial water content of the Moon decreased significantly between the time of its formation and the start of the LA. During the LA, increases in the D/H ratio and lowering of the Cl isotopic composition occurred, accompanied by an increase in water content. This suggests that the Moon's (and by extension Earth’s) initial volatiles were replenished ∼0.5 Ga after their formation, with final budgets reflecting a mixture of sources and delivery times. These findings are consistent with dynamic models that predict influxes of chemically and isotopically distinct asteroid populations at different times during the first ∼700 million years of solar-system evolution [17,53–57]. Future grain-scale age dating of lunar apatites from basalts outside the LA range combined with hydrogen and chlorine isotopic analyses on the same grains could further refine the timing of volatile fluxes to the Moon and Earth during the LA.

## METHODS

### Deriving source-region volatile contents from apatite volatile contents

The H_2_O and Cl concentrations in apatite shown in Figs. 1 and 2 were used to quantify lunar interior H_2_O and Cl contents. Quantification requires assumptions about (i) the partitioning of H_2_O and Cl between apatite and melt and (ii) a mantle-melting model leading to the crystallization of apatite. Quantifying apatite–melt partitioning of volatiles is not straightforward [42] and a comprehensive model of apatite–melt partition coefficients (*D* values, with *D_a_* the ratio in concentration by weight of element *a* in apatite and co-existing melt) for Cl and H_2_O is not available at present. Unlike the *D* values, apatite–melt volatile exchange coefficients *K*_d_ for OH–Cl (*K*Ap-Melt dOH–Cl  = $\frac{{\rm{C}{{\rm{l}}_{{\rm{Melt}}}}{\rm{*O}}{{\rm{H}}_{\rm{Ap}}}}}{{{\rm{O}}{{\rm{H}}_{{\rm{Melt}}}}{\rm{*C}}{{\rm{l}}_{{\rm{Ap}}}}}}$) do not vary substantially, at 0.06 ± 0.02 [42]. These exchange coefficients can be used to estimate quantitatively the water content of the mantle source if Cl contents in apatite and the corresponding parent melt are known [42]. We derived the Cl content of parent melt $X_{{\rm{Cl}}}^{{\rm{Parent\ Melt}}}$ by combining measured lunar whole-rock Nd and Ba contents with the constant Cl/Nd and Cl/Ba ratios observed in primitive lunar magmatic samples [48]. Parent melts were estimated to have formed by partially melting a mantle source to a melt fraction of 15%. Together, this leads to the following equation to derive the water content of mantle sources:

(1) \begin{eqnarray*} && {\rm{Wate}{\rm{r}_{{\rm{mantle\ source}}}}}\\ && = \frac{{F_{\rm{Source}}^{\rm{Partial\ Melting}}{\rm{*}}X_{\rm{H}2{\rm{O}}}^{{\rm{Ap}}}*X_{{\rm{Cl}}}^{{\rm{Parent\ Melt}}}}}{K_{{\rm{dOH}-{\rm{Cl}}}^{\rm{Ap} - {\rm{Melt}}}*X_{\rm{Cl}}^{\rm{Ap}}}}\ , \end{eqnarray*}

where $F_{{\rm{Source}}}^{{\rm{Partial\ Melting}}}$= 0.15, $K_{{\rm{dOH}}-{\rm{Cl}}}^{{\rm{Ap}} - {\rm{Melt}}}$=0.06, $X_{{\rm{H}}2{\rm{O}}}^{{\rm{Ap}}}$ is the H_2_O content of the apatite, and $X_{{\rm{Cl}}}^{{\rm{Ap}}}$ and $X_{{\rm{Cl}}}^{{\rm{Parent\ Melt}}}$ represent the Cl contents of apatite and parent melt, respectively. Combining published bulk-rock Nd and Ba contents of pre-LA KREEP basalt 72275 [58] and three post-LA high-Ti mare basalts (75055 [59], 10058 [60] and 10044 [61]), average values of Cl/Nd (0.216) and Cl/Ba (0.031) [48] and the above equation, the amounts of their mantle source water are yielded successively to be 1.3, 35.6, 24.4 and 38.5 ppm by the Nd-based calculations and 0.9, 16.3, 13.2 and 14.2 ppm by Ba-based estimates, respectively.

### Mass balance calculations

Equations (2) and (3) below describe the relationship between lunar interior water content and hydrogen isotopic composition δD before and after the LA, assuming 100% efficient addition of water to the Moon (no degassing) during the LA:

(2) \begin{eqnarray*} &&{{\rm{\delta }}{{\rm{D}}_{{\rm{post}} - {\rm{LA}}}}{\rm{*Wate}}{{\rm{r}}_{{\rm{post}} - {\rm{LA}}}}}\nonumber\\ &=& {\rm{\delta }}{{\rm{D}}_{{\rm{pre}} - {\rm{LA}}}}{\rm{\ *Wate}}{{\rm{r}}_{{\rm{pre}} - {\rm{LA}}}}{\rm{*}}\nonumber\\ &&\times\,\left( {1 - {\rm{Y}}} \right) + {\rm{\delta }}{{\rm{D}}_{{\rm{LA}}}}{\rm{*Wate}}{{\rm{r}}_{{\rm{LA}}}}*{\rm{Y}}, \end{eqnarray*}

where

(3) \begin{equation*} \begin{array}{@{}*{2}{l}@{}} {\rm{Y}}&{ = \displaystyle\frac{{\delta {{\rm{D}}_{{\rm{post}} - {\rm{LA}}}}*{\rm{Wate}}{{\rm{r}}_{{\rm{post}} - {\rm{LA}}}} - \delta {{\rm{D}}_{{\rm{pre}} - {\rm{LA}}}}*{\rm{Wate}}{{\rm{r}}_{{\rm{pre}} - {\rm{LA}}}}}}{{\delta {{\rm{D}}_{{\rm{LA}}}}*{\rm{Wate}}{{\rm{r}}_{{\rm{LA}}}} - \delta {{\rm{D}}_{{\rm{pre}} - {\rm{LA}}}}*{\rm{Wate}}{{\rm{r}}_{{\rm{pre}} - {\rm{LA}}}}}}}\\[12pt] {}&{\ = \displaystyle\frac{{\delta {{\rm{D}}_{{\rm{High}} - {\rm{Ti}}}}*{\rm{Wate}}{{\rm{r}}_{{\rm{High}} - {\rm{Ti}}}} - \delta {{\rm{D}}_{{\rm{KREEP}}}}*{\rm{Wate}}{{\rm{r}}_{{\rm{KREEP}}}}}}{{\delta {{\rm{D}}_{{\rm{OC}}}}*{\rm{Wate}}{{\rm{r}}_{{\rm{OC}}}} - \delta {{\rm{D}}_{{\rm{KREEP}}}}*{\rm{Wate}}{{\rm{r}}_{{\rm{KREEP}}}}}}} \end{array}. \end{equation*}

In Equation (3), High-Ti, KREEP and OC are high-Ti mare basalts (75055, 10058 and 10044), KREEP basalt (72275) and ordinary chondrite, respectively.

### Data availability

The authors declare that all relevant data supporting the findings of this study are available in the Supplementary data, available as Supplementary Data at *NSR* online.

## Supplementary Material

nwz033_Supplemental_File
