# Supplementary material for: Isotopic evidence for volatile replenishment of the Moon during the Late Accretion
Source: Natl Sci Rev. 2019 Mar 11;6(6):1247–54. doi: 10.1093/nsr/nwz033 (PMC8291620; doi:10.1093/nsr/nwz033)
Supplement: nwz033_Supplemental_File [file nwz033_supplemental_file.docx]

**SUPPLEMENTARY DATA**

**Isotopic evidence for volatile replenishment of the Moon during Late Accretion**

Yanhao Lin*, Wim van Westrenen*

*Corresponding authors. E-mail: [y.lin@vu.nl](mailto:xxxxx@xxxx.xxx), [w.van.westrenen@vu.nl](mailto:w.van.westrenen@vu.nl)

**This supplementary data file contains three Supplementary Tables, one figure, one Supplementary Note and a reference section.**

All data used in Figures. 1 and 2 in the main text are presented in the Supplementary Tables 1–3.

Supplementary Table 1. Compilation of literature data on H_2_O contents and D/H ratios of lunar apatites and crystallization ages for their host rocks.

| Sample (Ref.) | H_2_O (ppm) | 2σ | Weighted average* (ppm) | 2σ* | δD (‰) | 2σ | Weighted average* (‰) | 2σ* | Age (Ga) (Ref.) |
| --- | --- | --- | --- | --- | --- | --- | --- | --- | --- |
| Mg-Suite |  |  |  |  |  |  |  |  |  |
| 78235 [1] |  |  |  |  |  |  |  |  | 4.426 ± 0.065; U–Pb [12] |
| ,43_Ap1#1_1 | 209 | 3 | 768 | 550 | 203 | 238 | -27 | 98 |  |
| ,43_Ap1#1b_1 | 319 | 4 |  |  | -152 | 159 |  |  |  |
| ,43_Ap5_1 | 552 | 6 |  |  | 18 | 107 |  |  |  |
| ,43_Ap5#2_1 | 1636 | 19 |  |  | -14 | 125 |  |  |  |
| ,43_Ap5#4_1 | 1121 | 13 |  |  | -52 | 79 |  |  |  |
| 77215 [1] |  |  |  |  |  |  |  |  | 4.30 ± 0.02; Sm–Nd [13] |
| ,202_Ap2_1 | 453 | 14 | 896 | 520 | -205 | 163 | -281 | 49 |  |
| ,202_Ap4_1 | 1801 | 56 |  |  | -299 | 84 |  |  |  |
| ,202_Ap5_1 | 227 | 7 |  |  | -234 | 203 |  |  |  |
| ,202_Ap6_1 | 1482 | 46 |  |  | -271 | 95 |  |  |  |
| ,202_Ap9_1 | 904 | 28 |  |  | -264 | 132 |  |  |  |
| ,202_Ap8_1 | 512 | 16 |  |  | -384 | 175 |  |  |  |
| *76535 [1]* |  |  |  |  |  |  |  |  | 4.195 ± 0.024; Ar–Ar[14] |
| *,51_Ap1#1_1* | *9* | *0.3* | *11.0* | *8.0* | *3720* | *+1011/-1131* | *1734* | *1600* |  |
| *,51_Ap1#2_1* | *13* | *0.3* |  |  | *2055* | *+689/-783* |  |  |  |
| *,51_Ap1#3_1* | *8* | *0.3* |  |  | *3503* | *+1042/-1166* |  |  |  |
| *,51_Ap3#1_1* | *29* | *0.5* |  |  | *763* | *+440/-477* |  |  |  |
| *,51_Ap3#2_1* | *5* | *0.2* |  |  | *2336* | *+1733/-1897* |  |  |  |
| *,51_Ap3#3_1* | *3* | *0.2* |  |  | *11130* | *+3953/-3699* |  |  |  |
| *76535 [2]* |  |  |  |  |  |  |  |  |  |
| *,52_apt1* | *75* | *7* | *74* | *16* | *639* | *93* | *458* | *210* |  |
| *,56_#1* | *62* | *6* |  |  | *388* | *106* |  |  |  |
| *,56_#2* | *86* | *8* |  |  | *342* | *103* |  |  |  |
| *,56_#3 (3)* | *~200* |  |  |  | *~170* |  |  |  |  |
| 14303 [1] |  |  |  |  |  |  |  |  | ~4.1 ± 0.2; U–Pb, Rb–Sr and Ar–Ar [15–19] |
| ,205_Ap1_1 | 90 | 3 | 400 | 440 | -184 | 375 | -106 | 130 |  |
| ,205_Ap2a_16 | 547 | 17 |  |  | 35 | 216 |  |  |  |
| ,205_Ap2b_1 | 68 | 2 |  |  | -321 | 390 |  |  |  |
| ,205_Ap2c_1 | 1186 | 37 |  |  | -139 | 97 |  |  |  |
| ,205_Ap2d_1 | 110 | 4 |  |  | 110 | 295 |  |  |  |
| 79215 [4] |  |  |  |  |  |  |  |  | 3.9 ± 0.1; Ar–Ar [20] |
| AP2_6 | 25 | 8 |  |  | 1082 | 93 | 1055 | 190 |  |
| AP4_1 |  |  |  |  | 1234 | 104 |  |  |  |
| AP4_2 |  |  |  |  | 811 | 78 |  |  |  |
| AP4_3 |  |  |  |  | 1101 | 78 |  |  |  |
| 14305 [2,5] |  |  |  |  |  |  |  |  | 3.95 ± 0.17; Sm–Nd [21] |
| ,303_#390 | 80 | 10 | 80 | 7 | 238 | 72 | 236 | 200 |  |
| ,303_#391 | 80 | 10 |  |  | 341 | 53 |  |  |  |
| ,94_#322 | *-0.009* | *-0.001* |  |  | -96 | 60 |  |  |  |
| ,656_apt1 |  |  |  |  | 735 | 652 |  |  |  |
| ,656_apt3 #2 |  |  |  |  | 481 | 320 |  |  |  |
| ,656_apt3 #3 |  |  |  |  | -65 | 528 |  |  |  |
| ,656_apt3 #4 |  |  |  |  | 934 | 514 |  |  |  |
| ,656_apt4 |  |  |  |  | -107 | 459 |  |  |  |
| ,656_apt5 |  |  |  |  | -34 | 481 |  |  |  |
| ,656_apt6 |  |  |  |  | 608 | 418 |  |  |  |
| ,656_apt7 |  |  |  |  | -168 | 420 |  |  |  |
| *14161 [2]* |  |  |  |  |  |  |  |  |  |
| *,7069_triapt#1* | *162* | *49* | *174* | *30* | *114* | *66* | *200* | *140* | - |
| *,7069_triapt#2* | *189* | *57* |  |  | *165* | *62* |  |  |  |
| *,7373_apt3* | *174* | *52* |  |  | *323* | *70* |  |  | - |
| *15403 [2]* |  |  |  |  |  |  |  |  |  |
| *,71_apt1 #1* | *77* | *9* | *128* | *68* | *-597* | *79* | *-712* | *170* | - |
| *,71_apt1 #2* | *181* | *20* |  |  | *-724* | *48* |  |  |  |
| *,71_apt1 #3* | *129* | *14* |  |  | *-754* | *57* |  |  |  |
| *15404 [2]* |  |  |  |  |  |  |  |  |  |
| *,51_apt6 #1* | *58* | *6* | *106* | *50* | *-438* | *138* | *-581* | *96* | - |
| *,51_apt6 #2* | *46* | *6* |  |  | *-653* | *82* |  |  |  |
| *,55_apt1 #1* | *134* | *4* |  |  | *-592* | *432* |  |  | - |
| *,55_apt1 #2* | *267* | *9* |  |  | *-346* | *319* |  |  |  |
| *,55_apt2 #1* | *76* | *2* |  |  | *-352* | *492* |  |  |  |
| *,55_apt2 #2* | *99* | *3* |  |  | *-689* | *491* |  |  |  |
| *,55_apt2 #3* | *99* | *3* |  |  | *-547* | *491* |  |  |  |
| *,55_apt2 #4* | *69* | *2* |  |  | *-514* | *523* |  |  |  |
| High-Al Mare basalt |  |  |  |  |  |  |  |  |  |
| 14053 [5,6] |  |  |  |  |  |  |  |  | ~3.95 ± 0.04; Rb–Sr, Ar–Ar [22,23] |
| ,256_#315 | 860 | 140 | 713 | 150 | -172 | 24 | -111 | 57 |  |
| ,256_#318 | 640 | 100 |  |  | -215 | 96 |  |  |  |
| ,256_#387 | 870 | 140 |  |  | -175 | 37 |  |  |  |
| ,256_#388 | 930 | 150 |  |  | -202 | 23 |  |  |  |
| ,257_#320 | 670 | 110 |  |  | -202 | 31 |  |  |  |
| ,257_#321 | 570 | 100 |  |  | -179 | 40 |  |  |  |
| Pocket 1_Apt1 | 679 | 10 | 1190 | 700 | -15 | 47 |  |  |  |
| Pocket 2_Apt1 | 692 | 12 |  |  | -23 | 47 |  |  |  |
| Pocket 3_Apt1 | 2409 | 10 |  |  | -86 | 36 |  |  |  |
| Pocket 3_Apt3 | 1447 | 10 |  |  | -14 | 36 |  |  |  |
| Pocket 4_Apt2 | 721 | 11 |  |  | 53 | 49 |  |  |  |
| KREEP Basalt |  |  |  |  |  |  |  |  |  |
| 72275 [7] |  |  |  |  |  |  |  |  | 4.091 ± 0.049; Rb–Sr and Sm–Nd [7] |
| ,469_Ap2#1 | 50 | 2 | 270 | 130 | 184 | 316 | -113 | 78 |  |
| ,469_Ap3#1 | 46 | 2 |  |  | -105 | 293 |  |  |  |
| ,469_Ap4#1 | 160 | 6 |  |  | -139 | 249 |  |  |  |
| ,469_Ap5#1 | 80 | 3 |  |  | 15 | 228 |  |  |  |
| ,469_Ap6#1 | 123 | 5 |  |  | -81 | 460 |  |  |  |
| ,469_Ap6#2 | 249 | 10 |  |  | -118 | 386 |  |  |  |
| ,469_Ap7#1 | 262 | 11 |  |  | -129 | 213 |  |  |  |
| ,469_Ap8#1 | 174 | 7 |  |  | 187 | 254 |  |  |  |
| ,469_Ap9#1 | 368 | 14 |  |  | -143 | 162 |  |  |  |
| ,469_Ap10#1 | 536 | 21 |  |  | -180 | 172 |  |  |  |
| ,469_Ap10#2 | 858 | 34 |  |  | -157 | 133 |  |  |  |
| ,469_Ap12#1 | 344 | 14 |  |  | -277 | 211 |  |  |  |
| 15386 [7] |  |  |  |  |  |  |  |  | 3.912 ± 0.025; Rb–Sr and Sm–Nd [7] |
| ,45_Ap1#1 | 265 | 11 | 350 | 110 | 595 | 144 | 400 | 140 |  |
| ,45_Ap2#1 | 265 | 11 |  |  | 538 | 151 |  |  |  |
| ,45_Ap4#1 | 302 | 12 |  |  | 778 | 123 |  |  |  |
| ,45_Ap5#1 | 775 | 31 |  |  | 89 | 99 |  |  |  |
| ,45_Ap6#1 | 218 | 8 |  |  | 413 | 170 |  |  |  |
| ,45_Ap7#1 | 583 | 23 |  |  | 296 | 127 |  |  |  |
| ,45_Ap10#1 | 454 | 18 |  |  | 465 | 118 |  |  |  |
| ,45_Ap11#1 | 296 | 12 |  |  | 321 | 149 |  |  |  |
| ,45_Ap12#1 | 114 | 5 |  |  | 615 | 217 |  |  |  |
| ,45_Ap13#1 | 461 | 18 |  |  | 140 | 180 |  |  |  |
| ,45_Ap14#1 | 120 | 5 |  |  | 311 | 234 |  |  |  |
| ,45_Ap15#1 | 345 | 14 |  |  | 426 | 135 |  |  |  |
| QMDs |  |  |  |  |  |  |  |  |  |
| 14321 [2] |  |  |  |  |  |  |  |  |  |
| ,1047_apt1 #3 | 82 | 2 | 91 | 25 | 231 | 415 | 382 | 680 | ~3.98 ± 0.14; Sm–Nd, Rb–Sr and Ar-Ar [15,24,25] |
| ,1047_apt2 #2 | 137 | 3 |  |  | -118 | 364 |  |  |  |
| ,1047_apt4 | 93 | 2 |  |  | -327 | 439 |  |  |  |
| ,1047_apt6 #1 | 66 | 2 |  |  | 925 | 356 |  |  |  |
| ,1047_apt6 #2 | 76 | 2 |  |  | 790 | 317 |  |  |  |
| *77538 [2]* |  |  |  |  |  |  |  |  |  |
| *apt1* | 175 | 55 | 182 | 38 | *304* | *74* | *344* | *500* | *-* |
| *apt2* | 188 | 55 |  |  | *383* | *74* |  |  |  |
| High-Ti Mare Basalt |  |  |  |  |  |  |  |  |  |
| 75055 [8] |  |  |  |  |  |  |  |  | 3.82 ± 0.05; Rb–Sr and Ar–Ar [26–28] |
| ,55_Ap1#1 | 604 | 23 | 1125 | 380 | 621 | 35 | 795 | 150 |  |
| ,55_Ap1#2 | 1225 | 35 |  |  | 794 | 26 |  |  |  |
| ,55_Ap1#3 | 1241 | 30 |  |  | 968 | 27 |  |  |  |
| ,55_Ap1#4 | 1430 | 34 |  |  | 794 | 26 |  |  |  |
| 10058 [9] |  |  |  |  |  |  |  |  | 3.71 ± 0.04; Ar–Ar [29] |
| ,46_Ap1#1 | 1595 | 9 | 1351 | 130 | 724 | 74 | 823 | 100 |  |
| ,46_Ap1#2 | 1333 | 8 |  |  | 949 | 80 |  |  |  |
| ,46_Ap1#3 | 1118 | 7 |  |  | 1098 | 79 |  |  |  |
| ,46_Ap4#1 | 1387 | 8 |  |  | 678 | 84 |  |  |  |
| ,46_Ap4#2 | 1649 | 10 |  |  | 648 | 82 |  |  |  |
| ,46_Ap7#1 | 1111 | 7 |  |  | 735 | 86 |  |  |  |
| ,46_Ap3#1 | 1127 | 7 |  |  | 965 | 84 |  |  |  |
| ,46_Ap2#1 | 1338 | 8 |  |  | 929 | 79 |  |  |  |
| ,46_Ap2#2 | 1287 | 8 |  |  | 884 | 85 |  |  |  |
| ,46_Ap5#1 | 1568 | 9 |  |  | 618 | 77 |  |  |  |
| 10044 [4,8,10] |  |  |  |  |  |  |  |  | 3.722 ± 0.011; U–Pb [28] |
| Ap5_Pt1_1 | 1176 | 68 | 1178 | 100 | 622 | 48 | 734 | 56 |  |
| Ap5_Pt3_1 | 1455 | 84 |  |  | 623 | 49 |  |  |  |
| Ap5_Pt3_2 | 1379 | 79 |  |  | 657 | 63 |  |  |  |
| Ap5_Pt2_1 | 1622 | 94 |  |  | 682 | 46 |  |  |  |
| Ap6b_Pt2_1 | 1088 | 63 |  |  | 1005 | 46 |  |  |  |
| Ap6b_Pt2_2 | 1073 | 62 |  |  | 1013 | 58 |  |  |  |
| Ap11_Pt1_1 | 1078 | 62 |  |  | 697 | 49 |  |  |  |
| Ap5#2_1 | 1043 | 28 |  |  | 631 | 66 |  |  |  |
| Ap1#1_1 | 1161 | 32 |  |  | 617 | 64 |  |  |  |
| Ap1#2_1 | 1578 | 43 |  |  | 796 | 56 |  |  |  |
| Ap1#3_1 | 1394 | 38 |  |  | 710 | 61 |  |  |  |
| Ap1#5_1 | 1727 | 47 |  |  | 772 | 54 |  |  |  |
| Ap1b#1_1 | 1195 | 32 |  |  | 697 | 69 |  |  |  |
| Ap5#4_1 | 1424 | 37 |  |  | 675 | 63 |  |  |  |
| Ap7#1_1 | 837 | 22 |  |  | 533 | 85 |  |  |  |
| Ap7#2_1 | 1029 | 26 |  |  | 635 | 77 |  |  |  |
| Ap8#1_1 | 917 | 24 |  |  | 818 | 70 |  |  |  |
| Ap6b#3_1 | 1204 | 31 |  |  | 891 | 63 |  |  |  |
| ,12_#10 | 1105 | 29 |  |  | 932 | 31 |  |  |  |
| ,12_#1a | 728 | 27 |  |  | 954 | 27 |  |  |  |
| ,12_#1c | 1220 | 30 |  |  | 781 | 23 |  |  |  |
| ,644_#2 | 877 | 26 |  |  | 536 | 57 |  |  |  |
| ,644_#4a | 826 | 25 |  |  | 605 | 33 |  |  |  |
| ,644_#4b | 1155 | 29 |  |  | 702 | 28 |  |  |  |
| Low-Ti Mare Basalt |  |  |  |  |  |  |  |  |  |
| 15058 [9] |  |  |  |  |  |  |  |  | 3.40 ± 0.05; Ar–Ar and Rb–Sr [30–32] |
| ,20_Ap1#1 | 474 | 50 | 373 | 130 | 565 | 73 | 582 | 50 |  |
| ,20_Ap1#2 | 464 | 50 |  |  | 637 | 73 |  |  |  |
| ,20_Ap1#3 | 217 | 44 |  |  | 637 | 103 |  |  |  |
| ,20_Ap1#4 | 202 | 44 |  |  | 611 | 109 |  |  |  |
| ,20_Ap1#5 | 267 | 46 |  |  | 483 | 125 |  |  |  |
| ,20_Ap7#1 | 232 | 45 |  |  | 556 | 106 |  |  |  |
| ,20_Ap4#1 | 377 | 48 |  |  | 647 | 87 |  |  |  |
| ,20_Ap3#1 | 751 | 56 |  |  | 510 | 69 |  |  |  |
| 15555 [9] |  |  |  |  | 580.75 | 62 |  |  | ~3.35 ± 0.05; Ar–Ar and Rb–Sr [9,15] |
| ,991_Ap5#1 | 1202 | 57 | 2416 | 850 | 454 | 145 | 597 | 77 |  |
| ,991_Ap5#2 | 1405 | 60 |  |  | 682 | 115 |  |  |  |
| ,991_Ap2#1 | 3619 | 100 |  |  | 520 | 76 |  |  |  |
| ,991_Ap1#1 | 3188 | 92 |  |  | 578 | 56 |  |  |  |
| ,991_Ap1#2 | 3147 | 92 |  |  | 715 | 102 |  |  |  |
| ,991_Ap1#3 | 1940 | 70 |  |  | 632 | 63 |  |  |  |
| 12064 [10] |  |  |  |  | 596.8333333 | 64 |  |  | 3.18 ± 0.09; Rb–Sr [33] |
| Ap1_23 | 906 | 212 | 1183 | 310 | 880 | 72 | 887 | 43 |  |
| Ap1_23 | 783 | 184 |  |  | 831 | 74 |  |  |  |
| Ap2_Ap3_23 | 1274 | 299 |  |  | 1009 | 72 |  |  |  |
| Ap2_Ap3_23 | 1293 | 303 |  |  | 854 | 72 |  |  |  |
| Ap2_Ap3_23 | 1272 | 298 |  |  | 1008 | 71 |  |  |  |
| Ap2_Ap3_23 | 1048 | 246 |  |  | 960 | 73 |  |  |  |
| Ap2_Ap3_23 | 1323 | 310 |  |  | 849 | 72 |  |  |  |
| Ap2_Ap3_23 | 877 | 205 |  |  | 940 | 75 |  |  |  |
| App4_22 | 657 | 38 |  |  | 786 | 62 |  |  |  |
| App5_23 | 1219 | 70 |  |  | 955 | 45 |  |  |  |
| App5_23 | 125 | 73 |  |  | 947 | 45 |  |  |  |
| App6_23 | 1274 | 74 |  |  | 911 | 45 |  |  |  |
| App6abc_23 | 2320 | 134 |  |  | 798 | 47 |  |  |  |
| App7_23 | 2204 | 127 |  |  | 811 | 38 |  |  |  |
| 12039 [5,8,9] |  |  |  |  |  |  |  |  | 3.20 ± 0.05; Sm–Nd [34] |
| ,42_#286 | 2810 | 420 | 2982 | 480 | 680 | 30 | 776 | 54 |  |
| ,42_#287 | 2570 | 390 |  |  | 730 | 31 |  |  |  |
| ,42_#289 | 6050 | 740 |  |  | 672 | 35 |  |  |  |
| ,42_#309 | 5540 | 700 |  |  | 792 | 38 |  |  |  |
| ,42_#343 | 3040 | 440 |  |  | 472 | 63 |  |  |  |
| ,42_#344 | 3040 | 440 |  |  | 542 | 65 |  |  |  |
| ,42_#345 | 3590 | 510 |  |  | 391 | 62 |  |  |  |
| ,42_#347 | 5590 | 710 |  |  | 655 | 74 |  |  |  |
| ,42_#359 | 960 | 160 |  |  | 498 | 83 |  |  |  |
| ,42_#377 | 5450 | 700 |  |  | 875 | 40 |  |  |  |
| ,42_#378 | 5820 | 720 |  |  | 747 | 50 |  |  |  |
| ,42_#379 | 2740 | 410 |  |  | 1010 | 47 |  |  |  |
| ,42_#380 | 2960 | 430 |  |  | 892 | 45 |  |  |  |
| ,42_#4 | 1996 | 43 |  |  | 720 | 30 |  |  |  |
| ,42_#6 | 2379 | 47 |  |  | 830 | 31 |  |  |  |
| ,42_#17a | 2784 | 95 |  |  | 729 | 28 |  |  |  |
| ,42_#17b | 2916 | 57 |  |  | 698 | 28 |  |  |  |
| ,44_Ap2#1 | 2082 | 14 |  |  | 904 | 67 |  |  |  |
| ,44_Ap2#2 | 2410 | 16 |  |  | 967 | 65 |  |  |  |
| ,44_Ap1#1 | 2218 | 15 |  |  | 880 | 66 |  |  |  |
| ,44_Ap1#2 | 2968 | 19 |  |  | 767 | 63 |  |  |  |
| ,44_Ap1#3 | 1993 | 13 |  |  | 785 | 68 |  |  |  |
| ,44_Ap1#4 | 3798 | 23 |  |  | 812 | 60 |  |  |  |
| ,44_Ap1#5 | 1670 | 12 |  |  | 860 | 69 |  |  |  |
| ,44_Ap3#1 | 1930 | 13 |  |  | 889 | 67 |  |  |  |
| ,44_Ap3#2 | 1468 | 11 |  |  | 875 | 72 |  |  |  |
| ,44_Ap5#1 | 2378 | 15 |  |  | 822 | 65 |  |  |  |
| ,44_Ap6#1 | 1055 | 8 |  |  | 882 | 79 |  |  |  |
| ,44_Ap6#2 | 3180 | 20 |  |  | 991 | 61 |  |  |  |
| ,44_Ap8#1 | 3003 | 19 |  |  | 915 | 62 |  |  |  |
| 12040*** [5,8] |  |  |  |  |  |  |  |  | 3.21 ± 0.10; Ar–Ar [35] |
| ,211_#324 |  |  |  |  | 27 | 91 | -120 | 88 |  |
| ,211_#324_crack |  |  |  |  | -53 | 135 |  |  |  |
| ,211_#1 | 105 | 21 | 41 | 73 | 9 | 163 |  |  |  |
| ,211_#2 | 3 | 20 |  |  | 14 | 84 |  |  |  |
| ,211_#3 | 16 | 20 |  |  | -150 | 26 |  |  |  |
| Meteorite |  |  |  |  |  |  |  |  |  |
| MIL05035 [9] |  |  |  |  |  |  |  |  | 3.85 ± 0.10; Rb–Sr and Sm–Nd [36] |
| ,30_Ap11#1 | 4894 | 40 | 3990 | 990 | 237 | 39 | 311 | 87 |  |
| ,30_Ap11#3 | 4073 | 34 |  |  | 388 | 71 |  |  |  |
| ,30_Ap11#4 | 2719 | 24 |  |  | 417 | 99 |  |  |  |
| ,30_Ap1#1 | 3009 | 26 |  |  | 283 | 117 |  |  |  |
| ,30_Ap1#2 | 3107 | 26 |  |  | 157 | 47 |  |  |  |
| ,30_Ap1#3 | 2541 | 22 |  |  | 93 | 75 |  |  |  |
| ,30_Ap1#4 | 2044 | 19 |  |  | 157 | 101 |  |  |  |
| ,37_Ap7#1 | 7529 | 197 |  |  | 317 | 29 |  |  |  |
| ,37_Ap7#2 | 3337 | 110 |  |  | 443 | 32 |  |  |  |
| ,37_Ap3#1 | 4593 | 136 |  |  | 574 | 47 |  |  |  |
| ,37_Ap3#2 | 6061 | 166 |  |  | 341 | 49 |  |  |  |
| LAP 04841** [9] |  |  |  |  |  |  |  |  |  |
| ,19_Ap1#1 | 5717 | 196 | 5586 | 870 | 340 | 40 | 401 | 57 | 3.00 ± 0.02; [9] |
| ,19_Ap1#2 | 7364 | 251 |  |  | 278 | 72 |  |  |  |
| ,19_Ap1#3 | 7638 | 260 |  |  | 485 | 46 |  |  |  |
| ,19_Ap2#1 | 5735 | 196 |  |  | 403 | 55 |  |  |  |
| ,19_Ap2#2 | 6646 | 227 |  |  | 389 | 29 |  |  |  |
| ,19_Ap5#1 | 3745 | 130 |  |  | 515 | 38 |  |  |  |
| ,19_Ap5#2 | 3481 | 121 |  |  | 557 | 64 |  |  |  |
| ,19_Ap3#1 | 4765 | 164 |  |  | 385 | 61 |  |  |  |
| ,19_Ap3#2 | 5118 | 176 |  |  | 317 | 60 |  |  |  |
| ,19_Ap4#1 | 5686 | 195 |  |  | 329 | 53 |  |  |  |
| NWA 773 [7,11] |  |  |  |  |  |  |  |  |  |
| Ap6#1 | 1642 | 15 | 4755 | 1900 | -169 | 113 | 82 | 71 | 2.993 ± 0.032; Sm–Nd [37] |
| Ap6#2 | 2852 | 26 |  |  | -273 | 139 |  |  |  |
| Ap14#1 | 708 | 6 |  |  | 184 | 140 |  |  |  |
| Ap14#2 | 1210 | 11 |  |  | 23 | 90 |  |  |  |
| Ap9#1 | 1486 | 14 |  |  | -44 | 125 |  |  |  |
| Ap15#1 | 1568 | 14 |  |  | -54 | 124 |  |  |  |
| Ap16#1 | 517 | 5 |  |  | -64 | 145 |  |  |  |
| Ap16#2 | 1619 | 15 |  |  | 3 | 92 |  |  |  |
| Ap11#1 | 1132 | 11 |  |  | 38 | 98 |  |  |  |
| Ap2_1 | 1937 | 18 |  |  | -47 | 108 |  |  |  |
| Ap3#1 | 10528 | 96 |  |  | 224 | 60 |  |  |  |
| Ap3#2 | 9738 | 88 |  |  | 282 | 60 |  |  |  |
| Ap8#1 | 723 | 7 |  |  | -83 | 119 |  |  |  |
| Ap13#1 | 856 | 8 |  |  | 104 | 148 |  |  |  |
| Ap13#2 | 2535 | 23 |  |  | -105 | 79 |  |  |  |
| Ap13#3 | 1170 | 11 |  |  | -33 | 96 |  |  |  |
| Ap17#1 | 9442 | 86 |  |  | 309 | 59 |  |  |  |
| Ap17#3 | 11390 | 103 |  |  | 294 | 61 |  |  |  |
| Ap17#4 | 11892 | 108 |  |  | 189 | 75 |  |  |  |
| Ap22#1 | 5383 | 49 |  |  | 319 | 64 |  |  |  |
| Ap22#2 | 6889 | 63 |  |  | 144 | 63 |  |  |  |
| Ap21#1 | 7418 | 67 |  |  | 288 | 65 |  |  |  |
| NWA 4472 [11] |  |  |  |  |  |  |  |  |  |
| KREEP_ROI1_Ap1#1 | 2862 | 158 | 4298 | 710 | 9 | 92 | 126 | 190 | 3.936 ± 0.018; U–Pb [38] |
| KREEP_ROI1_Ap1#2 | 2849 | 157 |  |  | -8 | 113 |  |  |  |
| KREEP_ROI1_Ap1#3 | 4298 | 237 |  |  | -103 | 91 |  |  |  |
| KREEP_ROI1_Ap2#1 | 5458 | 302 |  |  | -95 | 69 |  |  |  |
| KREEP_ROI1_Ap2#2 | 3789 | 209 |  |  | -88 | 81 |  |  |  |
| KREEP_ROI1_Ap3#1 | 5145 | 284 |  |  | -92 | 64 |  |  |  |
| Matrix2_ROI5_Ap1#1 | 5338 | 295 |  |  | -176 | 72 |  |  |  |
| Matrix2_ROI5_Ap1#2 | 3387 | 187 |  |  | -12 | 84 |  |  |  |
| Matrix2_ROI5_Ap1#3 | 4523 | 250 |  |  | -243 | 150 |  |  |  |
| Matrix1_ROI6_Ap1#1 | 5282 | 292 |  |  | 741 | 54 |  |  |  |
| Matrix1_ROI6_Ap1#2 | 6438 | 356 |  |  | 486 | 62 |  |  |  |
| Matrix1_ROI6_Ap1#3 | 5759 | 318 |  |  | 579 | 58 |  |  |  |
| Matrix1_ROI6_Ap1#4 | 5349 | 295 |  |  | 904 | 44 |  |  |  |
| Matrix3_ROI11_Ap1#1 | 2305 | 127 |  |  | -66 | 99 |  |  |  |
| Matrix3_ROI11_Ap1#2 | 1775 | 98 |  |  | 26 | 140 |  |  |  |
| Kalahari 009 [11] |  |  |  |  |  |  |  |  |  |
| Section 1_Ap3#1 | 1105 | 5 | 1191 | 440 | -27 | 221 | -7 | 53 | 4.286 ± 0.095; Lu–Hf [39] |
| *Section 1_Ap4#1* | 525 | 3 |  |  | *451* | *213* |  |  |  |
| Section 1_Ap4#2 | 1245 | 6 |  |  | 51 | 239 |  |  |  |
| Section 1_Ap5#1 | 710 | 4 |  |  | 149 | 360 |  |  |  |
| Section 2_Ap1#1 | 1576 | 152 |  |  | -24 | 108 |  |  |  |
| Section 2_Ap1#2 | 1974 | 191 |  |  | -95 | 118 |  |  |  |
| Section 2_Ap1#3 | 645 | 62 |  |  | 54 | 160 |  |  |  |
| Section 2_Ap3#1 | 613 | 59 |  |  | 85 | 164 |  |  |  |
| Section 2_Ap5#2 | 2389 | 231 |  |  | -3 | 110 |  |  |  |
| Sayh al Uhaymir 169 [11] |  |  |  |  |  |  |  |  |  |
| ROI7_Ap1#1 | 1713 | 41 | 1957 | 640 | 138 | 96 | 61 | 160 | 3.92 ± 0.01; U–Pb [40] |
| ROI7_Ap1#2 | 2147 | 51 |  |  | 162 | 97 |  |  |  |
| ROI14_Ap1#1 | 1654 | 40 |  |  | 165 | 120 |  |  |  |
| ROI15_Ap1#1 | 1603 | 38 |  |  | -49 | 125 |  |  |  |
| ROI21_Ap1#1 | 3418 | 82 |  |  | -148 | 91 |  |  |  |
| ROI21_Ap1#2 | 1214 | 29 |  |  | 201 | 156 |  |  |  |
| Note: Data given in *italics* are for information only. As detailed in the Supplementary Information section, these data were not used to make scientific interpretations in the manuscript. | | | | | | | | | |
|  |  |  |  |  |  |  |  |  |  |
| * Values for weighted average ± error (2σ; 95% confidence level) calculated by the Isoplot/Ex_ver3 software [41] are given at assigned internal errors when MSWD<10, otherwise, they are given as assigned errors ± constant external errors. | | | | | | | | | |
|  |  |  |  |  |  |  |  |  |  |
| ** Exposure ages have not been determined for these samples. | | | | | | | | | |
| *** Sample may suffer from terrestrial contamination mentioned in Greenwood *et al.* [5]. | | | | | | | | | |

Supplementary Table 2. Compilation of Cl contents and δ^37^Cl ratios of lunar apatites and crystallization ages for their host rocks.

| Sample (Ref.) | Cl (ppm) | 2σ | Weighted average* (ppm) | 2σ* | δ^37^Cl (‰) | 2σ | Weighted average* (‰) | 2σ* | Age (Ga) (Ref.) |
| --- | --- | --- | --- | --- | --- | --- | --- | --- | --- |
| Mg-Suite |  |  |  |  |  |  |  |  |  |
| 78235 [42] |  |  |  |  |  |  |  |  | 4.43 ± 0.05; Sm–Nd [12] |
| Ap7a | 13689 | 205 | 11782 | 1000 | 31.2 | 1.6 | 31 | 2 |  |
| Ap7b | 13301 | 199 |  |  | 28.9 | 1.6 |  |  |  |
| Ap5a | 12322 | 185 |  |  | 29.6 | 1.6 |  |  |  |
| Ap5b | 10110 | 152 |  |  | 28.9 | 1.7 |  |  |  |
| Ap3a | 11435 | 171 |  |  | 29.1 | 1.2 |  |  |  |
| Ap3b | 10757 | 161 |  |  | 32.8 | 1.2 |  |  |  |
| Ap5c | 10897 | 163 |  |  | 33.8 | 1.2 |  |  |  |
| 76535 [3,42] |  |  |  |  |  |  |  |  | 4.195 ± 0.024; Ar–Ar [14] |
| ,56 | ~18000 |  |  |  | ~27.8 |  |  |  |  |
| Ap3aCl | 13163 | 164 | 13293 | 230 | 31.1 | 1.2 | 32 | 2 |  |
| Ap3bCl | 13428 | 167 |  |  | 32.1 | 1.3 |  |  |  |
| 79215 [3,4,42] |  |  |  |  |  |  |  |  | 3.9 ± 0.1; Ar–Ar [20] |
| ,51 | ~7800 |  |  |  | ~32.5 |  |  |  |  |
| ,51_#1 | 8253 |  |  |  | 32.7 | 1.6 |  |  |  |
| Ap13a | 7165 | 523 | 7303 | 170 | 29.3 | 0.9 | 29.7 | 2 |  |
| Ap13b | 7331 | 535 |  |  | 26.6 | 0.9 |  |  |  |
| Ap13c | 7036 | 514 |  |  | 26.9 | 0.9 |  |  |  |
| Ap13d | 7209 | 526 |  |  | 28.1 | 0.9 |  |  |  |
| Ap13e | 7293 | 533 |  |  | 27.6 | 0.9 |  |  |  |
| Ap1 | 6349 | 464 |  |  | 25.1 | 1.8 |  |  |  |
| Ap1 | 7561 | 552 |  |  | 31.7 | 1.6 |  |  |  |
| Ap3aCl | 7241 | 123 |  |  | 34.8 | 2 |  |  |  |
| Ap3b | 7345 | 124 |  |  | 36.3 | 2 |  |  |  |
| Ap2aCl | 7605 | 94 |  |  | 30 | 2 |  |  |  |
| Ap12Cl | 7169 | 89 |  |  | 33.8 | 2 |  |  |  |
| 12013 [3] |  |  |  |  |  |  |  |  | ~4.0 ± 0.1; U–Pb [15,45] |
| ,148_#1 | ~22000 |  |  |  | ~24,5 |  | 25 |  |  |
| ,148_#2 | ~16000 |  |  |  | ~26 |  |  |  |  |
| 14305 [3] |  |  |  |  |  |  |  |  | 3.95 ± 0.17; Sm–Nd [21] |
| ,94_#1 | ~2100 |  |  |  | ~27.5 |  | 30 |  |  |
| ,94_#2 | ~1900 |  |  |  | ~32.5 |  |  |  |  |
| KREEP Basalt |  |  |  |  |  |  |  |  |  |
| 72275,491 [43] | ~12200 |  |  |  | ~24.5 |  |  |  | 4.091 ± 0.049; Rb–Sr and Sm–Nd [7] |
| 15386 [42] |  |  |  |  |  |  |  |  | 3.912 ± 0.025; Rb–Sr and Sm–Nd [7] |
| Ap5a | 3764 | 55 | 2489 | 1800 | 30.8 | 1.3 | 23 | 11 |  |
| Ap10a | 2720 | 40 |  |  | 22.5 | 1.5 |  |  |  |
| Ap2a | 987 | 14 |  |  | 14.1 | 2.2 |  |  |  |
| VHK basalt |  |  |  |  |  |  |  |  |  |
| 14304 [42] |  |  |  |  |  |  |  |  | ~4.0 ± 0.05; Sm–Nd and Rb–Sr [46] |
| Ap1a | 2862 | 66 | 4038 | 600 | 28.1 | 1.5 | 28 | 1 |  |
| Ap1b | 4084 | 94 |  |  | 29.4 | 1.4 |  |  |  |
| Ap2a | 4100 | 94 |  |  | 24.5 | 2.8 |  |  |  |
| Ap4a | 2453 | 56 |  |  | 27.9 | 2.5 |  |  |  |
| Ap4b | 4148 | 95 |  |  | 25.9 | 2.5 |  |  |  |
| Ap4c | 5878 | 88 |  |  | 27.8 | 2.4 |  |  |  |
| Ap4d | 4940 | 74 |  |  | 29 | 2.4 |  |  |  |
| Ap5a | 3779 | 57 |  |  | 31.7 | 2.5 |  |  |  |
| Ap5b | 3815 | 57 |  |  | 28.8 | 2.5 |  |  |  |
| Ap8 | 4331 | 65 |  |  | 28.5 | 2.5 |  |  |  |
| High-Ti Mare Basalt |  |  |  |  |  |  |  |  |  |
| 10044 [8,42] |  |  |  |  |  |  |  |  | 3.722 ± 0.011; U–Pb [28] |
| ,12_#1 | 365 | 104 | 296 | 15 | 9 | 3 | 6 | 4 |  |
| ,12_#2 | 292 | 11 |  |  | 6 | 3 |  |  |  |
| ,12_#3 | 300 | 11 |  |  | 2 | 4 |  |  |  |
| ,644_#1 | 250 | 10 | 1227 | 1900 | 11 | 3 | 13 | 3 |  |
| ,644_#2 | 3976 | 122 |  |  | 15 | 3 |  |  |  |
| ,644_#3 | 219 | 10 |  |  | 12 | 3 |  |  |  |
| ,644_#4 | 479 | 16 |  |  | 12 | 3 |  |  |  |
| Ap5b | 258 | 4 | 487 | 400 | 16.1 | 4 | 9 | 6 |  |
| Ap6Da | 209 | 3 |  |  | 6.8 | 4.5 |  |  |  |
| Ap6Ca | 375 | 5 |  |  | 10 | 3.3 |  |  |  |
| Ap6Cb | 1109 | 16 |  |  | 6.5 | 2 |  |  |  |
| 10058 [42] |  |  |  |  |  |  |  |  | 3.71 ± 0.04; Ar–Ar [29] |
| Ap5 | 493 | 14 | 914 | 550 | 4.9 | 3.7 | 6 | 2 |  |
| Ap4a | 643 | 19 |  |  | 2.2 | 2.6 |  |  |  |
| Ap4b | 421 | 12 |  |  | 5.3 | 3 |  |  |  |
| Ap6a | 2478 | 57 |  |  | 9.8 | 2.4 |  |  |  |
| Ap6b | 1139 | 26 |  |  | 5 | 2.7 |  |  |  |
| Ap3 | 594 | 14 |  |  | 5.6 | 3.1 |  |  |  |
| Ap3b | 632 | 14 |  |  | 9.9 | 3 |  |  |  |
| 70035 [42] |  |  |  |  |  |  |  |  | 3.75 ± 0.07; Ar–Ar [23] |
| Ap17 | 968 | 14 | 1030 | 420 | 14.4 | 2.1 | 13 | 2 |  |
| Ap17 | 1379 | 50 |  |  | 13.4 | 1.8 |  |  |  |
| Ap15 | 746 | 13 |  |  | 10.8 | 2.5 |  |  |  |
| 75055 [8] |  |  |  |  |  |  |  |  | 3.82 ± 0.05; Rb–Sr and Ar–Ar [26–28] |
| ,55_#1 | 398 | 14 | 407 | 66 | 8 | 3 | 8.5 | 6 |  |
| ,55_#2 | 485 | 16 |  |  | 6 | 3 |  |  |  |
| ,55_#3 | 334 | 12 |  |  | 14 | 3 |  |  |  |
| ,55_#4 | 410 | 14 |  |  | 6 | 3 |  |  |  |
| Low-Ti Mare Basalt |  |  |  |  |  |  |  |  |  |
| 12040 [8,43] |  |  |  |  |  |  |  |  | 3.21 ± 0.10; Ar–Ar [35] |
| ,211_#1 | 15884 | 489 | 7291 | 7600 | 17 | 3 | 15 | 4 |  |
| ,211_#2 | 2689 | 83 |  |  | 13 | 3 |  |  |  |
| ,211_#3 | 3388 | 104 |  |  | 14 | 4 |  |  |  |
| ,46 | ~8300 |  |  |  | ~17.2 |  |  |  |  |
| 12039 [3] |  |  |  |  |  |  |  |  | 3.20 ± 0.05; Sm–Nd [34] |
| ,42_#1 | 928 | 29 | 805 | 320 | 18 | 3 | 17 | 3 |  |
| ,42_#2 | 477 | 16 |  |  | 17 | 3 |  |  |  |
| ,42_#3 | 1157 | 36 |  |  | 17 | 3 |  |  |  |
| ,42_#4 | 665 | 21 |  |  | 16 | 3 |  |  |  |
| 15555 [42,43] |  |  |  |  |  |  |  |  | ~3.35 ± 0.05; Ar–Ar [9,15] |
| Ap1a | 3143 | 47 | 2431 | 1800 | 12.8 | 1.5 | 13 | 2 |  |
| Ap1b | 3306 | 50 |  |  | 13.8 | 1.5 |  |  |  |
| Ap5b | 847 | 13 |  |  | 12.7 | 2.1 |  |  |  |
| ,207 | ~5600 |  |  |  | ~13.1 |  |  |  |  |
| Meteorite |  |  |  |  |  |  |  |  |  |
| MIL05035 [8,44] |  |  |  |  |  |  |  |  | 3.85 ± 0.10; Rb–Sr and Sm–Nd [36] |
| #1 |  |  |  |  | -4 | 2 |  |  |  |
| #2 |  |  |  |  | ~6 |  |  |  |  |
| NWA 4472 [11] |  |  |  |  |  |  |  |  | 3.936 ± 0.018; U–Pb [38] |
| KREEP_ROI1_Ap1#1 | 3939 | 50 | 3378 | 570 | 19.7 | 2.5 | 15.7 | 3 |  |
| KREEP_ROI1_Ap1#2 | 4166 | 53 |  |  | 17.2 | 2.4 |  |  |  |
| KREEP_ROI1_Ap1#3 | 2509 | 33 |  |  | 19.3 | 3.5 |  |  |  |
| KREEP_ROI1_Ap1#4 | 4017 | 68 |  |  | 18.1 | 3.2 |  |  |  |
| KREEP_ROI1_Ap1#5 | 3597 | 60 |  |  | 18.8 | 3 |  |  |  |
| KREEP_ROI1_Ap2#1 | 2946 | 38 |  |  | 18.5 | 3.1 |  |  |  |
| Matrix1_ROI6_Ap1#1 | 2280 | 29 |  |  | 15.3 | 3.3 |  |  |  |
| Matrix1_ROI6_Ap1#2 | 2420 | 31 |  |  | 7.1 | 3.1 |  |  |  |
| Matrix1_ROI6_Ap1#3 | 2992 | 50 |  |  | 6.8 | 3.3 |  |  |  |
| Matrix1_ROI6_Ap1#4 | 2848 | 48 |  |  | 12.5 | 3.5 |  |  |  |
| Matrix3_ROI11_Ap1#1 | 5451 | 70 |  |  | 15.4 | 2.4 |  |  |  |
| Sayh al Uhaymir 169 [11] |  |  |  |  |  |  |  |  | 3.92 ± 0.01; U–Pb [40] |
| ROI7_Ap1#1 | 8078 | 118 | 8411 | 1000 | 8.4 | 2.4 | 9.2 | 2 |  |
| ROI7_Ap1#2 | 6268 | 91 |  |  | 9.2 | 2.5 |  |  |  |
| ROI7_Ap1#3 | 7687 | 112 |  |  | 8.9 | 2.5 |  |  |  |
| ROI7_Ap1#4 | 10051 | 129 |  |  | 5.3 | 2.2 |  |  |  |
| ROI7_Ap1#5 | 6464 | 83 |  |  | 6.6 | 2.4 |  |  |  |
| ROI14_Ap1#1 | 9842 | 143 |  |  | 10.9 | 2.4 |  |  |  |
| ROI14_Ap1#2 | 9828 | 126 |  |  | 11.5 | 2.3 |  |  |  |
| ROI14_Ap1#3 | 10014 | 146 |  |  | 10.9 | 2.4 |  |  |  |
| ROI21_Ap1#1 | 7476 | 109 |  |  | 12.2 | 2.5 |  |  |  |
| *Values for weighted average ± error (2σ; 95% confidence level) calculated by the Isoplot/Ex_ver3 software [41] are given at assigned internal errors when MSWD<10, otherwise, they are given as assigned errors ± constant external errors. | | | | | | | | | |
|  |  |  |  |  |  |  |  |  |  |
|  |  |  |  |  |  |  |  |  |  |

Supplementary Table 3. Compilation of δD and δ^37^Cl values for major chemical reservoirs in the solar system used in Figs. 1c and 2c.

| Object | | δ D (‰) | Ref. | δ ^37^Cl (‰) | Ref. |
| --- | --- | --- | --- | --- | --- |
| VSMOW | | 0 | 47 |  |  |
| Protosolar | | -865 ± 32 | 48 |  |  |
| Model early Earth | | -815 – -300 | 49 |  |  |
| Earth samples | | -218 – -44±19 | 50, 51 | -0.35 – +0.02 | 67 |
| Mars | | -180 – 5079 | 52 | -3.8 – +8.6 | 68 |
| CC | CI* | 79 ± 6*** | 53 | -0.78 – +1.21 | 67 |
|  | CM | -230 – 335*** |  |  |  |
|  | CR | 264 – 765*** |  |  |  |
|  | CO** | -50*** |  |  |  |
|  | CV** | 14*** |  |  |  |
| Tagish Lake - 11i | | 540*** |  |  |  |
| Tagish Lake - 11h | | 560*** |  |  |  |
| Tagish Lake - 5b | | 508*** |  |  |  |
| OC** | | 1619*** |  | -3.61 – +0.52 | 67 |
| EC | | -460 | 54 | -0.5 – +0.79 | 67 |
| Jupiter | | -856 ± 22 | 55 |  |  |
| Saturn | | -891 (^+48^_-29_) |  |  |  |
| Uranus | | -647 (^+225^_-96_) | 56 |  |  |
| Neptune | | -711 ± 64 |  |  |  |
| *Saturn icy moon* | |  |  |  |  |
| Enceladus | | 861 (^+963^_-449_) | 57 |  |  |
| *Oort Cloud Comets* | |  |  |  |  |
| 1P/Halley | | 1028 ± 71 | 58 |  |  |
| Hyakutake | | 861 ± 642 | 59 |  |  |
| Hale-Bopp | | 1118 ± 513 | 60 |  |  |
| C/2002 T7 (LINEAR) | | 605 ± 257 | 61 |  |  |
| 8P Tuttle | | 1625 ± 931 | 62 |  |  |
| 153P/Ikeya-Zhang | | < 605 | 63 |  |  |
| *Jupiter family Comet* | |  |  |  |  |
| 103P/Hartley 2 | | 33 ± 154 | 64 |  |  |
| 45P HMP | | < 284 | 65 |  |  |
| Comet 67P/CG | | 2403 ± 450 | 66 |  |  |
| * The range is based on two samples of Orgueil and one of Ivuna. | | | | |  |
| ** There values are for the most primitive members of their groups. | | | | |  |
| *** Bulk δD for objects used in Fig. 1c. | | |  |  |  |


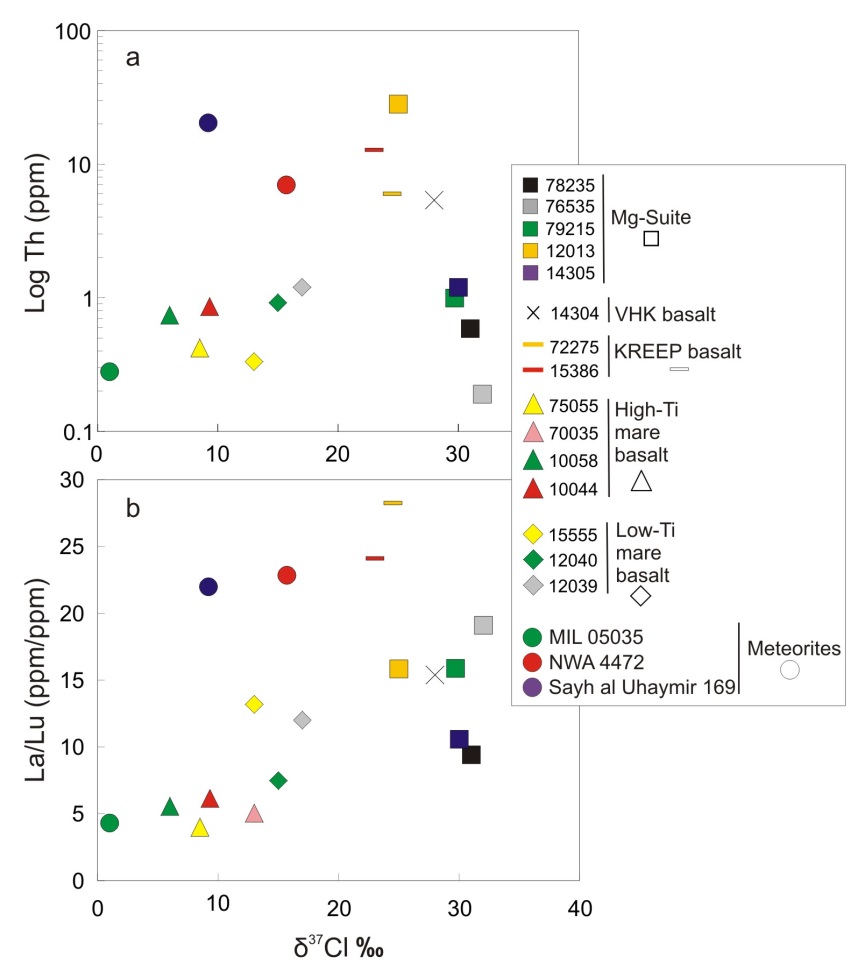


**Supplementary Figure 1**: Bulk rock Th (a) and La/Lu (b) versus δ^37^Cl of apatites.

**Supplementary Note:**

**Overview of samples including age information**

Mg-Suite samples:

*Sample 78235:*

This is a coarse-grained, heavily shocked norite composed of ∼51 vol.% orthopyroxene, ∼48 vol.% plagioclase, with accessory phases including phosphates, glasses and glass veins [30,71]. Although 78235 has experienced shock-induced deformation [71], it is thought to be one of the oldest highlands samples [30]. Recent age determinations seem to indicate a crystallization age around ∼4.35–4.44 Ga [73,74], making 78235 one of the oldest lunar samples. One of the youngest dates for this sample comes from ^40^Ar/^39^Ar dating, which yielded an age of 4.11 ± 0.02 Ga [75]. These results are consistent with work of Premo and Tatsumoto [12] who reported a U–Pb crystallization age of 4.426 ± 0.065 Ga, with a disturbance event dated at 3.93 ± 0.21 Ga. The younger ^40^Ar/^39^Ar and U–Pb dates likely record one or several impact events that affected this sample. In this study we use the U–Pb crystallization age of 4.426 ± 0.065 Ga [12] as a best estimate for the age of the apatites in 78235.

*Sample 77215:*

This is a brecciated norite with several lithic clasts which retain their primary igneous textures. Its modal mineralogy is approximately 41% orthopyroxene and 54% plagioclase with trace amounts of phosphates, ilmenite, spinel, and other minerals [76]. Apatite was analyzed in section 202 of sample 77215. Plagioclase in this sample has only partially been converted to maskelynite [1]. The sample contains low abundances of Ir and Ni attesting to its pristine nature [77]. Carlson *et al*. [13] define a crystallization age of 4.30 ± 0.02 Ga by Sm–Nd of minerals and whole rock, which is consistent with a previous Sm–Nd age determination [78]. We use this newly Sm–Nd age of 4.30 ± 0.02 Ga as a best estimate for the age of apatite in 77215.

*Sample 76535:*

Troctolite 76535 is a chemically pristine, un-shocked, coarse-grained plutonic rock [79]. It contains ∼40 to 50 vol.% plagioclase, ∼40 to 50 vol.% olivine, ∼4 vol.% orthopyroxene, accessory minerals and mesostasis [72,79,80]. This rock has a cumulate texture thought to be the result of slow cooling in a plutonic environment. It subsequently underwent a long period of sub-solidus re-equilibration and annealing, during which it was possibly affected by melt metasomatism creating symplectite assemblages [81,82], before being excavated by an impact which brought it onto the lunar surface. Sample 76535 can be considered as an end-member of the Mg-suite plutonic rocks. Crystallisation age estimates include a Pb–Pb age at ∼4.35 Ga [14]. Other published isotopic ages using the Sm–Nd, Rb–Sr and Ar–Ar systems show 4.295 ± 0.030, 4.308 ± 0.045 and 4.195 ± 0.024 Ga, respectively (summarized in Borg *et al.* [14]), because of different closure temperatures for these isotopic systems. Here we choose the recalculated Ar–Ar age of 4.195 ± 0.024 Ga [14] as the most likely age for apatite crystallization.

This sample may have been altered by post-crystallization and infiltrating exogenous metasomatism, which could have altered the primitive magmatic volatile contents of these apatites [1,81]. Apatites in troctolite 76535 display very low OH contents (< 50 ppm) and an extreme range in δD values between 763 +440/-477 ‰ and 11130 +3953/-3699 ‰ after spallation correction [1]. At such low OH contents, corrections for instrumental background and for spallation (including uncertainties in production rates of H and D) introduce large uncertainties, severely compromising the usefulness of these final data (see detailed explanation in the Supplementary Material of Barnes *et al*. [1]). For this reason, we show data for this sample in Supplementary Table 1 in italics, but we do not plot the data on Fig. 1.

*Sample 14303:*

This has been classified as a crystalline-matrix breccia, and identified as a strongly annealed clast-rich breccia from Fra Mauro in the lunar highlands [83]. It contains a large granite clast originally identified in section 14303,204 by Warren *et al*. [84], which is also represented in sample thin section 14303,205. Geochemically, the granite clast is pristine and mineralogically composed of ∼33 vol.% plagioclase, 32 vol.% K-feldspar, ∼23 vol.% silica, ∼11 vol.% pyroxene, and <1 vol.% ilmenite, with trace amounts of apatite, zircon, Fe–Ni metal, troilite, and olivine [84]. The coarseness of the graphic intergrowths and pyroxene exsolution lamellae supports an intrusive origin for the granite clast [84]. Estimates of sample age are summarized in the Lunar Sample Compendium [15]. Shih *et al*. [16,17] dated the granite clast using bulk-rock K–Ca and Rb–Sr methods and obtained an age of 3.95 ± 0.38 Ga, initially interpreted as a crystallisation age. This age is similar to a bulk-rock ^40^Ar/^39^Ar plateau age of 3.91 ± 0.04 Ga obtained by Kirsten *et al*. [18]. However, Meyer *et al*. [19] analyzed zircons in the granite clast and found U–Pb dates as old as 4.308 ± 0.004 Ga. This date therefore represents a minimum crystallisation age for the granite, with the younger K–Ca, Rb–Sr and 40Ar/39Ar ages at ∼3.9 Ga interpreted as the age of an impact event that formed the breccia. Here we use an average value of these ages of 4.1 ± 0.2 Ga.

*Sample 79215:*

This was found on the surface near Van Serg Crater as a feldspathic granulitic impactite, and was collected at the Apollo 17 site. It is holocrystalline, feldspar-rich with a granoblastic texture that was formed by a high-temperature metamorphic process of unknown origin [85]. On average the sample contains about 80 % plagioclase, 10 % olivine and 8 % pyroxene. The sample is low in KREEP element abundances, but high in meteoritic siderophiles. It is apparently an annealed aggregate of relict anorthositic and troctolitic cumulates, with an added meteoritic component. Recently, Hudgins *et al*. [20] used the bulk-rock Ar–Ar method and obtained a date of 3.9 ± 0.1 Ga, which is consistent with previous dating results [85,86]. In this study, we assume an apatite crystallization age of 3.9 ± 0.1 Ga.

*Sample 14305:*

This is a Fra Mauro breccia with a ~70% crystalline matrix breccia and ~30% clasts. 14305,303 is a whitlockite-bearing alkali anorthosite clast [84]. Texturally, it is considered a slightly annealed, but probably monomict breccia. The apatite is intergrown with whitlockite in this sample. One apatite grain fragment in the late-stage matrix brecciated region of 14305,94 was analyzed in Greenwood *et al*. [5]. The crystallization age of 14305 was suggested to be 3.92 Ga by the Ar–Ar releasing method [15]. However, various clasts in this breccia are older, and zircons are found to be extremely old (4.35 Ga). As for U–Pb dating of zircon (3.97–4.35 Ga) [88], there were about 3-group ages from three different genesis zircons, such as 4.35–4.26 Ga from Type-4 (fragmented grain with some of the original faces preserved) representing pristine crystallized age before impact, 4.22–4.30 Ga (zircon in a plagioclase grain) representing a same crystallization age with plagioclase, and 4.02–3.97 Ga from Type-3 (euhedral zircon with equal development of none of the original faces visible) showing recrystallization age after impact. Additionally, there was no apatite in 14305,17/103 with a U–Pb zircon age of 4.2 Ga. As noted by Greenwood *et al*. [5], apatite is the grain terminus, and could have formed later in the crystallization sequence resulting from impact remelting. Therefore, the crystallization age of apatite should be after 4.02 ± 0.01 – 3.97 ± 0.07 Ga, which is consistent with the whole-rock Sm–Nd isotopic age of 3.95 ± 0.17 Ga [21] and Ar releasing age of 3.92 ± 0.03 Ga [89] within error. Here, a whole-rock Sm–Nd age of 3.95 ± 0.17 Ga [21], consistent within error with an average value of these age data, is used.

*Sample 12013:*

This is a complex mixture of two polymict breccias. One is black and aphanitic and the other is mottled gray and white. The groundmass of the black breccia is a fine-grained intergrowth of plagioclase, pyroxene, ilmenite and phosphate minerals. The clast population is dominated by fragments of plagioclase, quartzofeldspathic rocks and norite. These clasts have not equilibrated with the groundmass. The chemical composition of the black breccia lithology is similar to KREEP. For further petrographic information, see the Lunar Sample Compendium [15]. Haines *et al*. [47] determined the age of various U–Th-rich minerals, including whitlockite, apatite, and zircon, by electron probe analysis at 4.0 ± 0.1 Ga. Subsequent radiogenic age dating summarized in Meyer [15] confirmed that the crystallization age of breccia 12013 is ~4 Ga, with whole rock measurements yielding 4.03 Ga by ^39^Ar–^40^Ar and 3.99 Ga by Rb–Sr. We assume 4.0 ± 0.1 Ga is a realistic age for the apatites in this sample.

High-Al mare basalts

*Sample 14053:*

14053 is an Al-rich mare basalt found perched on the side of a boulder at station C2 [90]. Petrographic information is summarized in Meyer [76]. 14053 has been dated with good precision by Rb–Sr (3.96 ± 0.04 Ga) [22] and Ar–Ar (3.94 ± 0.04 Ga) [23]. We use the average of these measurements, 3.95 ± 0.04 Ga, in this study. As noted by Greenwood *et al.* [5], a suggested petrologic history for this sample that involves 1) metasomatism on the lunar surface [82], followed by 2) implantation of solar wind hydrogen, and then 3) reheating in an impact event [91] could also be consistent with the low D/H measured here.

KREEP basalts

*Samples 72275 and 15386:*

72275 is a friable feldspathic breccia with an aphanitic matrix and several clasts [15]. It has a high abundance of KREEPy non-mare basalt. The matrix and many of the clasts contain significant Ir and Au abundances indicating meteorite contamination. 15386 is the largest sample of pristine KREEP basalt in the collection [76]. Crystallization ages of samples 72275 and 15386 are recalculated to be 4.091 ± 0.049 and 3.912 ± 0.025 Ga, respectively (weighted average calculated by combining Rb–Sr and Sm–Nd isochron dates using the Isoplot 3.0 add-in for Excel [41] and the revised ^87^Rb decay constant of 1.3968 x 10^-11^ a^-1^ [92], from data of Carlson and Lugmair [93], Nyquist *et al*. [94] and Shih *et al*. [95]) [7]. These recalculated ages are used in this study.

Clast-rich, Crystalline Matrix Breccia / Felsite

*Sample 14321:*

This, collected from the Fra Mauro Formation, is a clastic rock with a variety of lithic and microbreccia clasts [15]. Felsite sample 14321,1047 is well-known, consisting of clasts of graphically-intergrown quartz and K-feldspar, and resides in clast-rich impact breccia 14321 [84]. Sm–Nd, Rb–Sr and Ar–Ar whole-rock isotopic ages show a range from 4.12 to 3.83 Ga [15,24,25]. The age of the Fra Mauro Formation and Imbrium Event is about 3.85 ± 0.02 Ga [96]. The clasts in Apollo 14 breccias must necessarily be older than the event that created the breccias [15]. Therefore, we choose an average of the Sm–Nd, Rb–Sr and Ar–Ar ages (3.98 ± 0.14 Ga) as an estimate for the age of apatites from this sample.

High-Ti mare basalts

*Sample 75055:*

This sample consists of three pieces chipped from the side of a large boulder on the rim of Camelot Crater. This is a medium-grained ilmenite basalt that is slightly more aluminous and less titanium rich than other Apollo 17 basalts [97]. On the basis of its texture, the sample has been described as subophitic [98,99] with tabular plagioclase intergrown with subhedral to anhedral pyroxene and ilmenite laths. For further petrographic information, see the Lunar Sample Compendium [85]. Huneke *et al*. [26] and Kirsten *et al*. [27] showed a crystallization age of 3.82 ± 0.05 Ga based on Rb–Sr and Ar–Ar, which is consistent with a recent ^207^Pb/^206^Pb age of ~3.77 Ga [28]. Here we choose 3.82 ± 0.05 Ga as the crystallization age of sample 75055.

*Sample 10058:*

This is a low-K ilmenite basalt with a relatively coarse texture [76]. Here we use the Ar–Ar age of 3.71 ± 0.04 Ga [29].

*Sample 10044:*

This is a low-K ilmenite basalt that has a Ti content lower than typical ilmenite basalts from Apollo 11. On the basis of its texture, it was classified as coarse-grained porphyritic basalt [98] and microgabbro, which consists of subhedral to anhedral pyroxene, set in a matrix of plagioclase, anhedral pyroxene, and ilmenite, with minor apatite, spinel, silica, and symplectitic intergrowth [76]. Tartèse *et al*. [28] report a crystallization age of 3.722 ± 0.011 Ga from ^207^Pb/^206^Pb dating, consistent with a crystallization age of ~3.7 Ga reported by Meyer [76] based on older age determinations. We assume apatite crystallised at 3.722 ± 0.011 Ga.

*Sample 70035:*

This is a vesicular, medium-grained, high-Ti basalt, with reported ages varying from ~3.73 to ~3.82 Ga [85]. Here we assume the Ar–Ar age of 3.75 ± 0.07 Ga [23], consistent with the average of all reported ages.

Low-Ti mare basalts

*Sample 15058:*

This is a quartz-normative mare basalt with abundant elongate pyroxene crystals [49]. Its age is estimated at ~3.4 Ga [30]. We use an average age of 3.40 ± 0.05 Ga based on Ar–Ar age of 3.358 ± 0.025 Ga [31] and Rb–Sr age of 3.46 ± 0.04 Ga [32].

*Sample 15555:*

This contains olivine and pyroxene phenocrysts and is olivine-normative in composition. The sample has a subophitic, basaltic texture and there is little evidence for shock in the minerals [15]. Following Tartese *et al*. [9] we assume the sample age is 3.35 ± 0.05 Ga based on the average or reported ages summarized in Meyer [15].

*Sample 12064:*

This is a coarse-grained ilmenite basalt characterized by anhedral pyroxene crystals intergrown with anhedral plagioclase and rare subhedral plagioclase tablets, with sample ages clustering close to 3.2 Ga [30]. We adopted the Rb–Sr age of 3.18 ± 0.09 Ga [33] for this study.

*Sample 12039:*

This is a medium-grained pigeonite basalt/microgabbro, mainly composed of plagioclase and pyroxene with long needles of ilmenite and tridymite cutting across the plagioclase and pyroxene. Texturally, the sample ranges from porphyritic to subophitic to granular [76]. Consistent with the average of age determinations summarized in Meyer [75], we consider a Sm–Nd age of 3.20 ± 0.05 Ga [34] for this sample.

*Sample 12040:*

This is a coarse-grained olivine basalt with a high proportion of mafic minerals. Texturally, it is equigranular with an average grain size of 1 mm and the largest crystals being 3–4 mm in length [35]. It is mainly composed of olivine and pyroxene with minor plagioclase, ilmenite, chromite, troilite, metal, phosphates, and alkali feldspar [100]. For further petrographic information, see the Lunar Sample Compendium [76]. It has been successfully dated at about 3.2 Ga as summarized in Meyer [76]. Here we employ the Ar–Ar age of 3.21 ± 0.1 Ga reported by Compston *et al*. [35].

*Sample 14304:*

This is a clast-rich impact-melt breccia characterized by a wide range of mineral and lithic clast types in a recrystallized matrix [101]. The very high-K aluminous mare basalt clasts from 14304 yield an Ar–Ar age of ~3.95 Ga and Sm–Nd age of ~4.04 Ga [48]. Here we choose their average (4.0 ± 0.05 Ga).

Meteorites:

Detailed petrographic, mineralogical, chemical and geochronological information of the lunar meteorites used for this study, including MIL 05035, LAP 04841, NWA 773, NWA 4472, Kalahari 009 and Sayh al Uhaymir 169, is summarized in Tartèse *et al*. [11] and the Lunar Meteorite Compendium [102].

For MIL 05035, Rb–Sr and Sm–Nd internal isochron ages of MIL 05035 range from 3.9 ± 0.04 to 3.8 ± 0.01 Ga [36]. The average of 3.85 ± 0.10 Ga is used in Fig. 1.

Most of the ages obtained on LAP samples are in the range 3.04–2.93 Ga by ^207^Pb/^206^Pb, U–Pb ^40^Ar–^39^Ar, Rb–Sr and Sm–Nd isochron methods, and a weighted mean age of 3.00 ± 0.02 Ga for LAP samples was summarized in Tartèse *et al*. [9]. Therefore, we assumed 3.00 ± 0.02 Ga as the crystallization age of LAP 04841.

NWA 773 shows a consistent crystallization age of ~3.0 Ga within error by different dating methods, including Ar–Ar, Sm–Nd and Pb/Pb isochron dating as summarized in Tartèse *et al*. [11]. Here we use a whole-rock and mineral separate age of 2.993 ± 0.032 Ga obtained by Borg *et al*. [37].

In meteorite NWA 4472, apatite chronology shows ~4.0–3.9 Ga by U–Pb dating, with the exception of one older matrix apatite grain dated at ~4.34 Ga [38]. Merrillite chronology of NWA 4472 yields 3.93–3.99 Ga. Arai *et al*. [103] found similarly young 3.929–3.933 Ga zircon ages in the paired NWA 4485 stone, which they interpreted to indicate a thermal resetting event happened at ~3.93 Ga. Therefore, we choose 3.94 ± 0.018 Ga from apatite U–Pb dating [38] for this study.

In Kalahari 009, phosphate Lu–Hf dating yielded a crystallization age of 4.286 ± 0.095 Ga [39]. This crystallization age is used in this study.

In Sayh al Uhaymir 169, zircon U–Pb dating results yield two peak ages at 3.920 ± 0.013 Ga and 3.914 ± 0.007 Ga [40]. We assumed 3.92 ± 0.01 Ga as an appropriate crystallization age for this study.

*Samples 77538, 14161, 15403 and 15404:*

To date, to our knowledge no age information is available for Apollo samples 77538, 14161, 15403 and 15404. We list apatite measurements in italics in the subsequent tables, but cannot discuss these samples further in this particular manuscript.

**Supplementary References**

1. Barnes, JJ, Tartèse, R and Anand, M. *et al.* The origin of water in the primitive Moon as revealed by the lunar highlands samples. *Earth Planet Sci Lett* 2014; **390**: 244–52.
2. Robinson, KL, Barnes, JJ and Nagashima, K. *et al.* Water in evolved lunar rocks: Evidence for multiple reservoirs. *Geochim Cosmochim Acta* 2016; **188**: 244–60.
3. Boyce, JW, Guan, Y and Treiman, AH. *et al.* Volatile components in the moon; Abundances and isotope ratios of Cl and H in lunar apatites. *Lunar Planet Sci Conf* 2013; **44**: 2851.
4. Treiman, AH, Boyce, JW and Greenwood, JP. *et al.* D-poor hydrogen in lunar mare basalts assimilated from lunar regolith. *Am Mineral* 2016; **101**: 1596–603.
5. Greenwood, JP, Itoh, S and Sakamoto, N. *et al.* Hydrogen isotope ratios in lunar rocks indicate delivery of cometary water to the Moon. *Nat Geosci* 2011; **4**: 79–82.
6. Pernet-Fisher, JF, Howarth, GH and Liu, Y. *et al.* Estimating the lunar mantle water budget from phosphates: Complications associated with silicate-liquid-immiscibility. *Geochim Cosmochim Acta* 2014; **144**: 326–41.
7. Tartèse, R, Anand, M and McCubbin, FM. *et al.* Apatites in lunar KREEP basalts: The missing link to understanding the H isotope systematics of the Moon. *Geology* 2014; **42**: 363–6.
8. Boyce, JW, Treiman, AH and Guan, Y. *et al.* The chlorine isotope fingerprint of the lunar magma ocean. *Sci Adv* 2015; **1**: 1–8.
9. Tartèse, R, Anand, M and Barnes, JJ. *et al.* The abundance, distribution, and isotopic composition of Hydrogen in the Moon as revealed by basaltic lunar samples: Implications for the volatile inventory of the Moon. *Geochim Cosmochim Acta* 2013; **122**: 58–74.
10. Barnes, JJ, Franchi, IA and Anand, M. *et al.* Accurate and precise measurements of the D/H ratio and hydroxyl content in lunar apatites using NanoSIMS. *Chem Geol* 2013; **337–338**: 48–55.
11. Tartèse, R, Anand, M and Joy, KH. *et al.* H and Cl isotope systematics of apatite in brecciated lunar meteorites Northwest Africa 4472, Northwest Africa 773, Sayh al Uhaymir 169, and Kalahari 009. *Meteorit Planet Sci* 2014; **49**(12): 2266–89.
12. Premo, WR and Tatsumoto, M. U–Th–Pb isotopic systematics of lunar norite 78235. *Lunar Planet Sci Conf* 1991; **21**: 89–100.
13. Carlson, RW, Borg, L and Gaffney, A. *et al.* Rb–Sr, Sm–Nd, Lu–Hf isotope systematics of norite 77215: Refining the age and duration of lunar crust formation. *Lunar Planet Sci Conf* 2013; **44**: 1621.
14. Borg, L, Connelly, J and Cassata, W. *et al.* Evidence for widespread magmatic activity at 4.36 Ga in the lunar highlands from young ages determined on troctolite 76535. *Lunar Planet Sci Conf* 2013; **44**: 1563.
15. Meyer, C. *Lunar Sample Compendium* (NASA, Houston, TX). 2009.
16. Shih, CY, Nyquist, LE and Wiesmann, H. K–Ca chronology of lunar granites. *Geochim Cosmochimi Acta* 1993; **57**: 4827–41.
17. Shih, CY, Nyquist, LE and Bogard, DD. *et al.* K–Ca and Rb–Sr dating of two lunar granites: Relative chronometer resetting. *Geochim Cosmochim Acta* 1994; **58**: 3101–16.
18. Kirsten T, Deubner, J and Horn, P. *et al.* The rare gas record of Apollo 14 and 15 samples. *Proceedings Lunar and Planetary Science Conference* 1972; **3**: 1865–89.
19. Meyer, C, Williams, IS and Compston, W. Uranium-lead ages for lunar zircons: Evidence for a prolonged period of granophyre formation from 4.32 to 3.88 Ga. *Meteorit Planet Sci* 1996; **31**: 370–87.
20. Hudgins, JA, Spray, JG and Kelley, SP. *et al.* A laser probe Ar/Ar and INAA investigation of four Apollo granulitic breccias. *Geochim Cosmochim Acta* 2008; **72**: 5781–98.
21. Shih CY, Nyquist, LE and Bogard, DD. *et al.* Geochronology and petrogenesis of Apollo 14 very high potassium mare basalts. *Proc Lunar Planet Sci Conf* 1986; **16**: D214–D228.
22. Papanastassiou, DA and Wasserburg, GJ. Rb–Sr ages of igneous rocks from the Apollo 14 mission and the age of the Fra Mauro formation. *Earth Planet Sci Lett* 1971; 12: 36–48.
23. Stettler, A, Eberhardt, P and Geiss, J *et al.* ^39^Ar–^40^Ar ages and ^37^Ar–^38^Ar exposure ages of lunar rocks. *Proc Lunar Planet Sci Conf* 1973; **4**: 1865–88.
24. Turner, G, Huneke, JC and Podosek, FA. *et al.* ^40^Ar–^39^Ar ages and cosmic ray exposure ages of Apollo 14 samples. *Earth Planet Sci Lett* 1971; **12**: 19–35.
25. York, D, Kenyon, WJ and Doyle, RJ. ^40^Ar–^39^Ar ages of Apollo 14 and 15 samples. *Proc Lunar Planet Sci Conf* 1972; **3**: 1613–22.
26. Huneke, JC, Jessberger, EK and Podosek, FA. *et al.* Ar/Ar measurements in Apollo 16 and 17 samples and the chronology of metamorphic and volcanic activity in the Taurus-Littrow region. *Proc Lunar Planet Sci Conf* 1973; **2**: 1725–56.
27. Kirsten, T, Horn, P, and Heymann, D. Chronology of the Taurus-Littrow region 1: Ages of two major rock types from the Apollo 17 site. *Earth Planet Sci Lett* 1973; **20**: 125–30.
28. Tartèse, R, Anand, M and Delhaye, T. NanoSIMS Pb/Pb dating of tranquillityite in high-Ti lunar basalts: Implications for the chronology of high-Ti volcanism on the Moon. *Am Mineral* 2013; **98**: 1477–86.
29. Guggisberg, S, Eberhardt, P and Geiss, J. *et al.* Classification of the Apollo-11 mare basalts according to ^39^Ar–^40^Ar ages and petrological properties. *Proc Lunar Planet Sci Conf* 1979; **10**: 1–39.
30. Meyer, C. *Lunar Sample Compendium* (NASA, Houston, TX). 2010.
31. Husain, L. ^40^Ar–^39^Ar chronology and cosmic-ray exposure age of the Apollo 15 sample. *J Geophys Res* 1974; **79**: 2588–606.
32. Birck, JL, Fourcade, S and Allègre, CJ. ^87^Rb–^86^Sr age of rocks from the Apollo 15 landing site and significance of internal isochrones. *Earth Planet Sci Lett* 1975; **26**: 29–35.
33. Papanastassiou, DA and Wasserburg, GJ. Lunar chronology and evolution from Rb–Sr studies of Apollo 11 and 12 samples. *Earth Planet Sci Lett* 1971; **11**: 37–62.
34. Nyquist, LE, Shih, C–Y and Wooden, JL. *et al.* The Sr and Nd isotopic record of Apollo 12 basalts: Implications for lunar geochemical evolution. *Proc Lunar Planet Sci Conf* 1979; **10**: 77–114.
35. Compston, W, Berry, H and Vernon, MJ. *et al.* Rubidium-strontium chronology and chemistry of lunar material from the Ocean of Stroms. *Proc Lunar Planet Sci Conf* 1971; **2**: 1471–85.
36. Nyquist, LE, Shih, CY and Reese, Y. *et al.* Sm–Nd and Rb–Sr ages for MIL 05035: Implications for surface and mantle sources. *Lunar Planet Sci Conf* 2007; **38**: 1702.
37. Borg, LE, Gaffney, AM and Shearer, CK. Mechanisms for incompatible-element enrichment on the Moon deduced from the lunar basaltic meteorite Northwest Africa 032. *Geochim Cosmochim Acta* 2009; **73**: 3963–80.
38. Joy, KH, Burgess, R and Hinton, R *et al.* Petrogenesis and chronology of lunar meteorite Northwest Africa 4472: A KREEPy regolith breccia from the Moon. *Geochim Cosmochim Acta* 2011; **75**: 2420–52.
39. Sokol, AK, Fernandescd, VA and Schulz, T. *et al.* Geochemistry, petrology and ages of the lunar meteorites Kalahari 008 and 009: New constraints on early lunar evolution. *Geochim Cosmochim Acta* 2008; **72**: 4845–73.
40. Liu, D, Jolliff, BL and Zeigler, RA. *et al.* Comparative zircon U–Pb geochronology of impact melt breccias from Apollo 12 and lunar meteorite SaU 169, and implications for the age of the Imbrium impact. *Earth Planet Sci Lett* 2012; **319–320**: 277–86.
41. Ludwig, KR. ISOPLOT 3.00: A Geochronological Toolkit for Microsoft Excel. Berkeley Geochronology Center, Berkeley, CA. 2003.
42. Barnes, JJ, Tartèse, R and Anand, M. *et al.* Early degassing of lunar urKREEP by crust-breaching impact(s). *Earth Planet Sci Lett* 2016; **447**: 84–94.
43. Sharp, ZD, Shearer, CK and Mckeegan, KD. *et al.* The chlorine isotope composition of the Moon and implications for an anhydrous mantle. *Science* 2010; **329**: 1050–3.
44. Potts, NJ, Barnes, JJ and Tartèse, R. *et al*. Chlorine isotopic compositions of apatite in Apollo 14 rocks: Evidence for widespread vapor-phase metasomatism on the lunar nearside ~4 billion years ago. *Geochim Cosmochim Acta* 2018; **230**: 46–59.
45. Hui, H, Peslier, AH and Zhang, Y. *et al.* Water in lunar anorthosites and evidence for a wet early Moon. *Nat Geosci* 2013; B: 177–80.
46. Wang, Y, Guan, Y and Hsu, W. *et al.* Water content, chlorine and hydrogen isotope compositions of lunar apatite. *Annual Meteorit Society Meeting* 2012; **75**: 5170.
47. Haines, EL, Albee, AL and Chodos, AA. *et al.* Uranium-bearing minerals of lunar rock 12013. *Earth Planet Sci Lett* 1971; **12**: 145–54.
48. Shih, C-Y, Nyquist, LE and Bogard, DD. *et al.* Geochronology of high-K aluminous mare basalt clasts from Apollo 14 breccia 14304. *Geochim Cosmochim Acta* 1987; **51**: 3255–71.
49. De Laeter, JR, Bohlke, JK and De Bievre P. *et al*. Atomic weights of the elements: review 2000, (IUPAC Technical report). *Pure and Applied Chem* 2003; **75**: 683–800.
50. Geiss, J and Gloeckler, G. Abundances of deuterium and helium-3 in the protosolar cloud. *Space Sci Rev* 1998; **84**: 239.
51. Sharp, ZD. Nebular ingassing as a source of volatiles to the Terrestrial planets. *Chem Geol* 2017; **448**: 137–50.
52. Hallis, LJ, Huss, GR and Nagashima, K. *et al.* Evidence for primordial water in Earth’s deep mantle. *Science* 2015; **350**: 336–9.
53. Lécuyer, C, Gillet, P and Robert, F. The hydrogen isotope composition of seawater and the global water cycle. *Chem* Geol 1998; **145**: 249.
54. Usui, T, Alexander, CMO’D and Wang, J. *et al.* Origin of water and mantle-crust interactions on Mars inferred from hydrogen isotopes and volatile element abundances of olivine-hosted melt inclusions of primitive shergottites. *Earth Planet Sci Lett* 2012; **357-358**: 119–29.
55. Alexander, CMO’D, Bowden, R and Fogel, ML. *et al.* The Provenances of Asteroids, and Their Contributions to the Volatile Inventories of the Terrestrial Planets. *Science* 2012; **337**: 721–3.
56. Javoy, M. The major volatile elements of the Earth: Their origin, behavior, and fate. *Geophys Res Lett* 1997; **24**: 177–80.
57. Lellouch, E, Bézard, B and Fouchet, T. *et al.* The deuterium abundance in Jupiter and Saturn from ISO-SWS observations. *Astron Astrophys* 2001; **670**: 610–22.
58. Feuchtgruber, H, Lellouch, E and Bézard, B. *et al.* Detection of HD in the atmospheres of Uranus and Neptune: a new determination of the D/H ratio. *Astron Astrophys* 1999; **341**: L17–L21.
59. Waite Jr, JH, Lewis, WS and Magee, BA. *et al.* Liquid water on Enceladus from observations of ammonia and ^40^Ar in the plume. *Nature* 2009; **460**: 487–90.
60. Eberhardt, P, Reber, M and Krankowsky, D. *et al.* The D/H and ^18^O/^16^O ratios in water from comet Halley. *Astron. Astrophys*.1995; **302**: 301.
61. Bockelée-Morvan, D, Gautier, D and Lis, DC. *et al.* Deuterated water in comet C/1996 B2 (Hyakutake) and its implications for the origin of comets. *Icarus* 1998; **133**: 147–62.
62. Meier, R, Owen, TC and Matthews, HE. *et al.* A determination of the HDO/H_2_O ratio in comet C/1995 O1 (Hale-Bopp). *Science* 1998; **279**: 842.
63. Hutsemékers, D, Manfroid, J and Jehin, E., *et al.* The ^16^OH/^18^OH and OD/OH isotope ratios in comet C/2002 T7 (LINEAR). *Astron Astrophys* 2008; **490**: L31.
64. Villanueva, GL, Mumma, MJ and Bonev, BP. *et al*., A sensitive search for deuterated water in comet 8P/Tuttle. *Astrophys. J.* 2009; **690**: L5.
65. Biver, N, Bockelée-Morvan, D and Crovisier, J. *et al.* Radio wavelength molecular observations of comets C/1999 T1 (McNaught-Hartley), C/2001 A2 (LINEAR), C/2000 WM1 (LINEAR) and 153P/Ikeya-Zhang. *Astron Astrophys* 2006; **449**: 1255.
66. Hartogh, P, Lis, DC and Bockelée-Morvan, D. *et al.* Ocean-like water in the Jupiter-family comet 103P/Hartley 2. *Nature* 2011; **478**: 218–20.
67. Lis, DC, Biver, N and Bockelée-Morvan, D. *et al.* A herschel study of D/H in water in the Jupiter-family Comet 45P/Honda-Mroks-Pajdušáková and prospects for D/H measurements with CCAT. *Earth Planet Astrophys* 2013; **774**: L3.
68. Altwegg, K, Balsiger, H and Bar-Nun, A. *et al*. 67P/Churyumov-Gerasimenko, a Jupiter family comet with a high D/H ratio. *Science* 2015; **347**(6220): 1261952.
69. Sharp, ZD, McCubbin, FM and Shearer, CK. The chlorine isotope composition of chondrites and Earth. *Geochim Cosmochim Acta* 2013; **107**: 189–204.
70. Sharp, Z, Williams, J and Shearer, C. *et al.* (2016) The chlorine isotope composition of Martian meteorites 2. Implications for the early solar system and the formation of Mars. *Meteorit Planet Sci* 2016; **51**: 2111–26.
71. McCallum, IS and Mathez, EA. Petrology of noritic cumulates and a partial melting model for the genesis of Fra Mauro basalts. *Proc Lunar Planet Sci Conf* 1975; **6**: 395–414.
72. Dymek, RF, Albee, AL and Chodos, AA. Comparative petrology of lunar cumulate rocks of possible primary origin: Dunite 72415, troctolite 76535, norite 78235, and anorthosite 62237. *Proc Lunar Planet Sci Conf* 1975; **6**: 301–41.
73. Andreasen, R, Simmons, ST and Righter, M. *et al.* Lutetium–hafnium and samarium–neodymium systematics of Apollo 17 sample 78235: Age and the importance of thermal neutron fluence on the lutetium–hafnium system. *Lunar Planet Sci Conf* 2013; **44**: 2887.
74. Edmunson, J, Borg, LE and Nyquist, LE. *et al.* A combined Sm–Nd, Rb–Sr, and U–Pb isotopic study of Mg-suite norite 78238: Further evidence for early differentiation of the Moon. *Geochim Cosmochim Acta* 2009; **73**: 514–27.
75. Aeschlimann, U, Eberhardt, P and Geiss, J. *et al.* On the age of cumulate norite 78236: an ^39^Ar-^40^Ar study. *Lunar Planet Sci Conf* 1982; **13**: 1–2.
76. Meyer, C. *Lunar Sample Compendium* (NASA, Houston, TX). 2011.
77. Chao, ECT, Minkin, JA and Thompson, CL. The petrology of 77215, a noritic impact ejecta breccia. *Proc Lunar Planet Sci Conf* 1976; **7**: 2287–308.
78. Nakamura N, Tatsumoto, M and Nunes, PD. 4.4 b.y.-old clast in Boulder 7, Apollo 17: A comprehensive chronological study by U–Pb, Rb–Sr, and Sm–Nd methods. *Proc Lunar Planet Sci Conf* 1976; **7**: 2309–33.
79. Warren, PH. A concise compilation of petrologic information on possibly pristine nonmare Moon rocks. *Am Mineral* 1993; 78: 360–76.
80. McCallum, IS and Schwartz, JM. Lunar Mg suite: Thermobarometry and petrogenesis of parental magmas. *J Geophys Res* 2001; **106**: 27969–83.
81. Elardo, SM, McCubbin, FM and Sheare, JCK. Chromite symplectites in Mg-suite troctolite 76535 as evidence for infiltration metasomatism of a lunar layered intrusion. *Geochim Cosmochim Acta* 2012; **87**: 154–77.
82. Neal, CR and Taylor, LA. Evidence for metasomatism of the lunar highlands and the origin of whitlockite. *Geochim Cosmochim Acta* 1991; **55**: 2965–80.
83. Chao, ECT, Minkin, JA and Best, JB. Apollo 14 breccias: General characteristics and classification. *Proc Lunar Planet Sci Conf* 1972; **3**: 645–59.
84. Warren, PH, Taylor, GJ and Keil, K. *et al.* Petrology and geochemistry of two large granite clasts from the Moon. *Earth Planet Sci Lett* 1983; **64**: 175–85.
85. Meyer, C. *Lunar Sample Compendium* (NASA, Houston, TX). 2008.
86. McGEE, JJ, Bence, AE and Eichhorn, G. *et al.* Feldspathic granulite 79215: Limitations on T-ƒ_O2_ conditions and time of metamorphism. *Proc Lunar Planet Sci Conf* 1978; **9**: 743–72.
87. Oberli, F, Huneke, JC, and Wasserburg, GJ. U–Pb and K–Ar systematics of cataclysm and precataclysm lunar impactites. *Lunar Planet Sci Conf* 1979; **10**: 940–2.
88. Nemchin, AA, Pidgeon, RT and Whitehouse, MJ. *et al.* SIMS study of zircons from Apollo 14 and 17 breccias: Implications for the evolution of lunar KREEP. *Geochimica et Cosmochimica Acta* 2008; **72**: 668–89.
89. Eugster O, Eberhardt, P and Geiss, J. *et al.* Cosmic ray exposure histories of Apollo 14, Apollo 15 and Apollo 16 rocks. *Proc Lunar Planet Sci Conf* 1984; **14**: B498–B512.
90. Swann, GA. Geology of the Apollo 14 landing site in the Fra Mauro Highlands. *U.S.G.S. Prof. Paper* 1977; 880.
91. Taylor, LA, Patchen, A, Mayne, RG and Taylor, D. *et al.* The most reduced rock from the moon, Apollo 14 basalt 14053: Its unique features and their origin. *Am Mineral* 2004; 89: 1617–24.
92. Rotenberg, E, Davis, DW and Amelin, Y. *et al.* Determination of the decay-constant of ^87^Rb by laboratory accumulation of ^87^Sr. *Geochim Cosmochim Acta* 2012; **85**: 41–57.
93. Carlson, RW and Lugmair, GW. Sm–Nd constraints on early lunar differentiation and the evolution of KREEP. *Earth Planet Sci Lett* 1979; **45**: 123–32.
94. Nyquist, LE, Bansal, BM and Wiesmann, H. Rb–Sr ages and initial ^87^Sr/^86^Sr for Apollo 17 basalts and KREEP basalt 15386. *Proc Lunar Planet Sci Conf* 1975; **6**: 1445–65.
95. Shih, CY, Nyquist, LE and Bansal, BM. *et al.* (1992) Rb–Sr and Sm–Nd chronology of an Apollo 17 KREEP basalt. *Earth Planet Sci Lett* 1992; **108**: 203–15.
96. Stöffler, D and Ryder, G. Stratigraphy and isotopic ages of lunar geologic units: Chronological standard for the inner solar system. *Space Sci Rev* 2001; **96**: 9–54.
97. Rhodes, JM, Wiesmann, H and Rodgers, KV. *et al.* Chemistry, classification, and petrogenesis of Apollo 17 mare basalts. *Proc Lunar Planet Sci Conf* 1976; **7**: 1467–89.
98. McGee, PE, Warner, JL and Simonds, CH. Introduction to the Apollo Collections. Part 1: Lunar Igneous Rocks (NASA, Washington, DC). 1977.
99. Dymek, RF, Albee, AL and Chodos, AA. Paper presented at the *6th Lunar and Planetary Science Conference*, Houston, TX. 1975.
100. French, BM, Walter, LS and Heinrich, KFJ. *et al.* Compositions of Major and Minor Minerals in Five Apollo 12 Crystalline Rocks (NASA, Greenbelt, MD). 1972.
101. Meyer, C. *Lunar Sample Compendium* (NASA, Houston, TX). 2007.
102. Righter, K. *Lunar Meteorite Compendium*. 2010.
103. Arai, T, Yoshitake, M and Tomiyama, T. *et al.* Support for a prolonged KREEP magmatism: U–Pb age dating of zircon and baddeleyite in lunar meteorite NWA 4485. *Lunar Planet Sci Conf* 2010; **41**: 2379.
